# Reducing the energy cost of walking with low assistance levels through optimized hip flexion assistance from a soft exosuit

**DOI:** 10.1038/s41598-022-14784-9

**Published:** 2022-06-29

**Authors:** Jinsoo Kim, Brendan T. Quinlivan, Lou-Ana Deprey, Dheepak Arumukhom Revi, Asa Eckert-Erdheim, Patrick Murphy, Dorothy Orzel, Conor J. Walsh

**Affiliations:** 1 John A. Paulson School of Engineering and Applied Sciences, Harvard University, Boston, 02134 USA; 2 School of Life Sciences, Swiss Federal Institute of Technology (EPFL), Lausanne, Switzerland; 3 Department of Mechanical Engineering, Boston University, Boston, 02215 USA; 4 College of Health and Rehabilitation Sciences: Sargent College, Boston University, Boston, 02215 USA

**Keywords:** Engineering, Physiology, Health care, Biophysics, Neuroscience

## Abstract

As we age, humans see natural decreases in muscle force and power which leads to a slower, less efficient gait. Improving mobility for both healthy individuals and those with muscle impairments/weakness has been a goal for exoskeleton designers for decades. In this work, we discover that significant reductions in the energy cost required for walking can be achieved with almost 50% less mechanical power compared to the state of the art. This was achieved by leveraging human-in-the-loop optimization to understand the importance of individualized assistance for hip flexion, a relatively unexplored joint motion. Specifically, we show that a tethered hip flexion exosuit can reduce the metabolic rate of walking by up to 15.2 ± 2.6%, compared to locomotion with assistance turned off (equivalent to 14.8% reduction compared to not wearing the exosuit). This large metabolic reduction was achieved with surprisingly low assistance magnitudes (average of 89 N, ~ 24% of normal hip flexion torque). Furthermore, the ratio of metabolic reduction to the positive exosuit power delivered was 1.8 times higher than ratios previously found for hip extension and ankle plantarflexion. These findings motivated the design of a lightweight (2.31 kg) and portable hip flexion assisting exosuit, that demonstrated a 7.2 ± 2.9% metabolic reduction compared to walking without the exosuit. The high ratio of metabolic reduction to exosuit power measured in this study supports previous simulation findings and provides compelling evidence that hip flexion may be an efficient joint motion to target when considering how to create practical and lightweight wearable robots to support improved mobility.

## Introduction

Walking is an extremely efficient motion. As humans, we optimize many aspects of our gait, such as step length^1^ and arm swing^2^, to minimize the energetic cost of walking. Any deviations from natural, healthy walking such as load carriage or pathological gait increases the energetic cost of walking. For example, stroke survivors can develop hemiparetic gait, a slow, asymmetric, and highly inefficient gait that stems from diminished paretic ankle push-off power^3,4^. Additionally, gait slowing and freezing of gait are common features of Parkinson’s disease, which result in energetically suboptimal walking with reduced step length and cadence^5,6^.

Reducing the metabolic cost of walking of both healthy individuals and those with gait impairments has been a goal for exoskeleton designers for decades. Preliminary success of reducing metabolic cost relative to natural, healthy walking didn’t occur until 2013, when Malcolm et al*.* focused on assisting a single joint motion, ankle plantarflexion, with a pneumatically powered ankle exoskeleton while tuning the control profile to that one joint motion^7^. Following Malcolm et al*.*’s work, many research groups began focusing on assisting single-joint motions and demonstrated larger and larger metabolic benefits, primarily targeting ankle plantarflexion^8–20^ or hip extension^21–26^.

Subsequently, in an attempt to further increase these metabolic benefits, research groups began applying assistance to multiple joint motions with a single exoskeleton including combining both ankle plantarflexion and hip extension with hip flexion^26–38^. Despite the success of these multi-joint systems that in-part assist hip flexion, relatively few researchers have stepped back and tried to isolate and understand the metabolic and biomechanical effects of hip flexion assistance including what assistance profiles (i.e., timings and magnitude of assistance) are most beneficial. A large number of publications have combined hip flexion assistance with another joint motions^26–38^ but only three publications^34,39,40^ have studied hip flexion assistance independently.

Of the exoskeletons that have assisted hip flexion, either independently or with another joint movement, most implemented one of only two assistive torque profiles: (i) an assistive torque profile based on the biological hip joint torque^32,39–42^ or (ii) an assistive torque profile based on the electromyography (EMG) signals from the hip muscles^41,43^. Comparatively, hip extension and ankle plantarflexion timings have been thoroughly investigated^7–11,21,22,44^. The lack of research dedicated to hip flexion is especially surprising as multiple simulation studies have indicated that it may be more economical to assist hip flexion compared to other joint motions^45,46^. In one of the few studies that did investigate the timing of hip flexion assistance, Young et al*.* found that a muscle inspired profile reduced metabolic cost and muscle activity more than the biological torque inspired profile^41^. Young et al*.* also investigated onset timing for assisting hip flexion independently but the resolution of assistance changes was limited with a pneumatic exoskeleton^34^.

One recently developed technique to identify an individualized, optimal profile of assistance is human-in-the-loop (HIL) optimization, where the value of a physiological objective (e.g., metabolic cost) is estimated in real-time (with a human in the loop) and used to identify optimal device parameters for each participant^8,11,22,44,47–52^. The motivation of this approach is to tackle high inter-participant variability in response to any given assistance, implying that assistive profiles that help one participant may hinder another. Since the HIL optimization was first introduced^48^, many research groups have tried to identify the optimal assistance of ankle plantarflexion^8,11,44,49,53^, hip extension^22^, and multi-joint systems^47,51,52^ during walking^8,22,44,47–53^ and running^11^. Studies have used a variety of optimization algorithms such as brute-force searches like a discrete step or continuous sweep, gradient descent, and gradient-free stochastic optimization methods like covariance matrix adaptation evolution strategy (CMA-ES) or Bayesian optimization.

**Figure 1:**
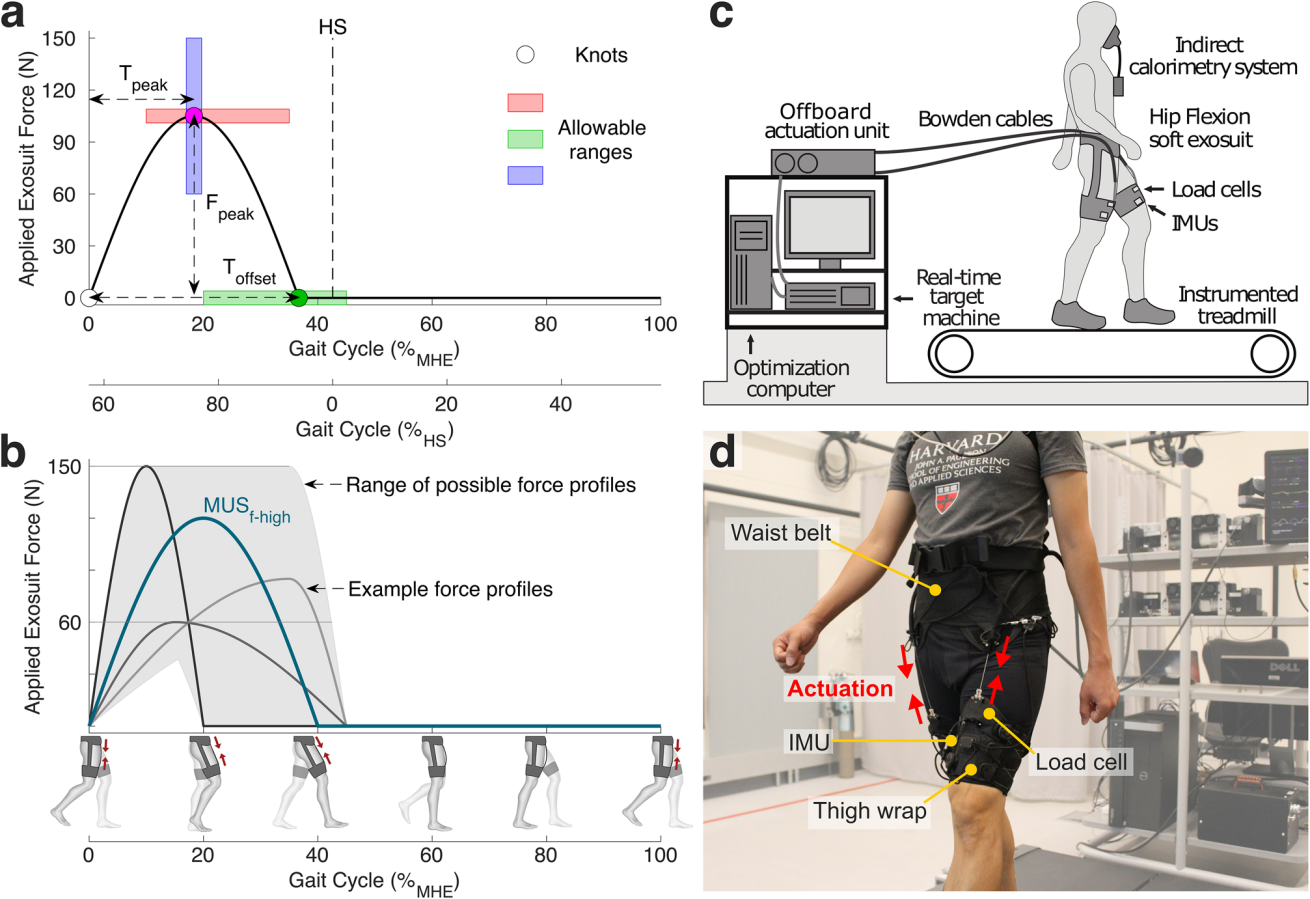
Experimental setup & HIL Parameters. (**a**) Parametrization of hip flexion force profile. There are three knot points (onset, peak, and offset), and a sinusoidal curve is used between knot points. The shaded region represents an allowable range for each parameter. %_MHE_ means the gait cycle segmented by a maximum hip extension (MHE), whereas %_HS_ means the gait cycle segmented by a heel strike (HS). A vertical dashed line indicates the heel strike. (**b**) Examples of possible assistance profiles and a fixed muscle inspired profile (${MUS}_{f-high}$). (**c**) Experimental setup with an off-board actuator. (**d**) Detailed view of components of hip flexion exosuit.

The aim of this work was to study the physiological and biomechanical effects of assisting hip flexion with a lightweight, soft exosuit. We explored how to individualize hip flexion assistance using HIL optimization, parameterized by peak force magnitude (${F}_{peak}$), peak force timing (${T}_{peak}$), and offset force timing (${T}_{offset}$) (Fig. 1a). This study can be separated into three key investigations. First, to verify the need for individualized assistance, we evaluated metabolic cost reduction associated with the individualized assistance profile (${OPT}_{f-opt}$), compared with that of a fixed assistance profile (${MUS}_{f-high}$) that was based on the muscle activation inspired profile in^41,43^ and had a constant force (Fig. 1b). Second, to examine the effects of assistance force magnitude and to validate one dimension of the optimization we performed a discrete step search of peak force magnitude (${F}_{peak}$) with the timing parameters (${T}_{peak}$ and ${T}_{offset}$) constant based on the optimal profile determined from the HIL method. Finally, to gain further insight into the effects of assistance timing on hip flexion assistance, we compared the optimal assistance profile (${OPT}_{f-opt}$) to both a biological torque inspired profile (${TOR}_{f-opt}$) such as those implemented in^32,39–42^ and a muscle activation inspired profile (${MUS}_{f-opt}$), all at the same force magnitude determined from the HIL method. Together, we believe these three investigations will provide insight into the best way to provide hip flexion assistance and the potential opportunity for a lightweight, portable hip flexion exosuit to aid both healthy individuals and those with gait impairments.

## Results

### Human-in-the-loop optimization

We conducted optimization tests with eight male participants walking on a treadmill at 1.25 m s^−1^, using a high-bandwidth, force-controlled off-board actuator with a bilateral hip flexion exosuit (Fig. 1c and d). The use of this off-board actuator enabled rapid alteration of assistance profiles during the optimization process without having to worry about the effects of carrying the additional weight. We used the CMA-ES algorithm to estimate the individualized, energetically optimal assistance profile (${OPT}_{f-opt}$) for each participant (Supplementary Methods). We then subsequently assessed reductions in metabolic cost during walking with optimal assistance, compared with walking with assistance turned off ($ASSIST \: OFF$) and without wearing the exosuit ($NO \: EXO$). Furthermore, additional active conditions with different assistance timing and magnitudes were included to gain further insight into the effectiveness of HIL optimization and the biomechanical effects of independently assisting hip flexion.

**Figure 2:**
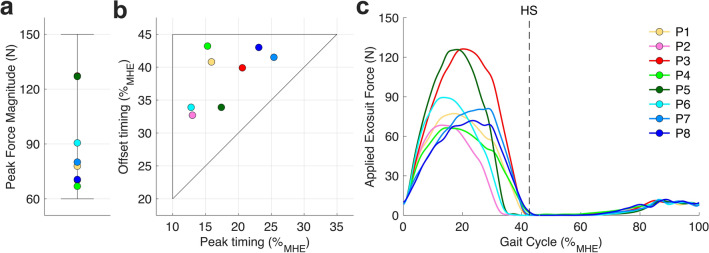
Optimal parameters. Optimal (**a**) peak magnitude (${F}_{peak}$) and (**b**) peak timing (${T}_{peak}$) and offset timing (${T}_{offset}$) in %_MHE_ gait cycle, segmented by a maximum hip extension (MHE), for each participant. The black solid boundary represents the parameter space. Participants 3 and 5 (P3 and P5) needed high assistance magnitude, whereas the rest of the participants needed low to medium assistance magnitude. P2, P5, and P6 had early peak and early offset optimal timings. P1 and P4 had early peak but late offset optimal timings. P3, P7, and P8 had late peak and late offset optimal timings. (**c**) The resultant optimal force profile applied to each participant. Each color represents one participant. The vertical dashed line indicates a heel strike.

As shown in Fig. 2, the resultant optimal force profiles varied substantially across the 8 participants and no two participants converged to the same profile (Supplementary Table S1), confirming the importance of individualized assistance. Optimal peak force magnitude varied from 67 to 127 N (0.10 to 0.23 Nm kg^−1^) with an average of 89 N (0.15 Nm kg^−1^). Optimal peak force timing varied from 12.8%_MHE_ to 25.4%_MHE_ with an average of 17.9%_MHE_ (MHE: Maximum Hip Extension). Finally, optimal offset force timing varied from 32.7%_MHE_ to 43.2%_MHE_ with an average of 38.6%_MHE_. Additionally, the optimal peak torque magnitude (${\tau }_{opt}$; Nm) positively correlated with participants’ body weight ($BW$; kg). An extra kg of body weight was associated with a 0.32 Nm increase in the optimal peak torque magnitude on average (${\tau }_{opt}=0.32\cdot BW-12.76$; *P* < 0.05), meaning that participants with larger body weight need higher assistance.

### Optimal profile vs. fixed profile

**Figure 3:**
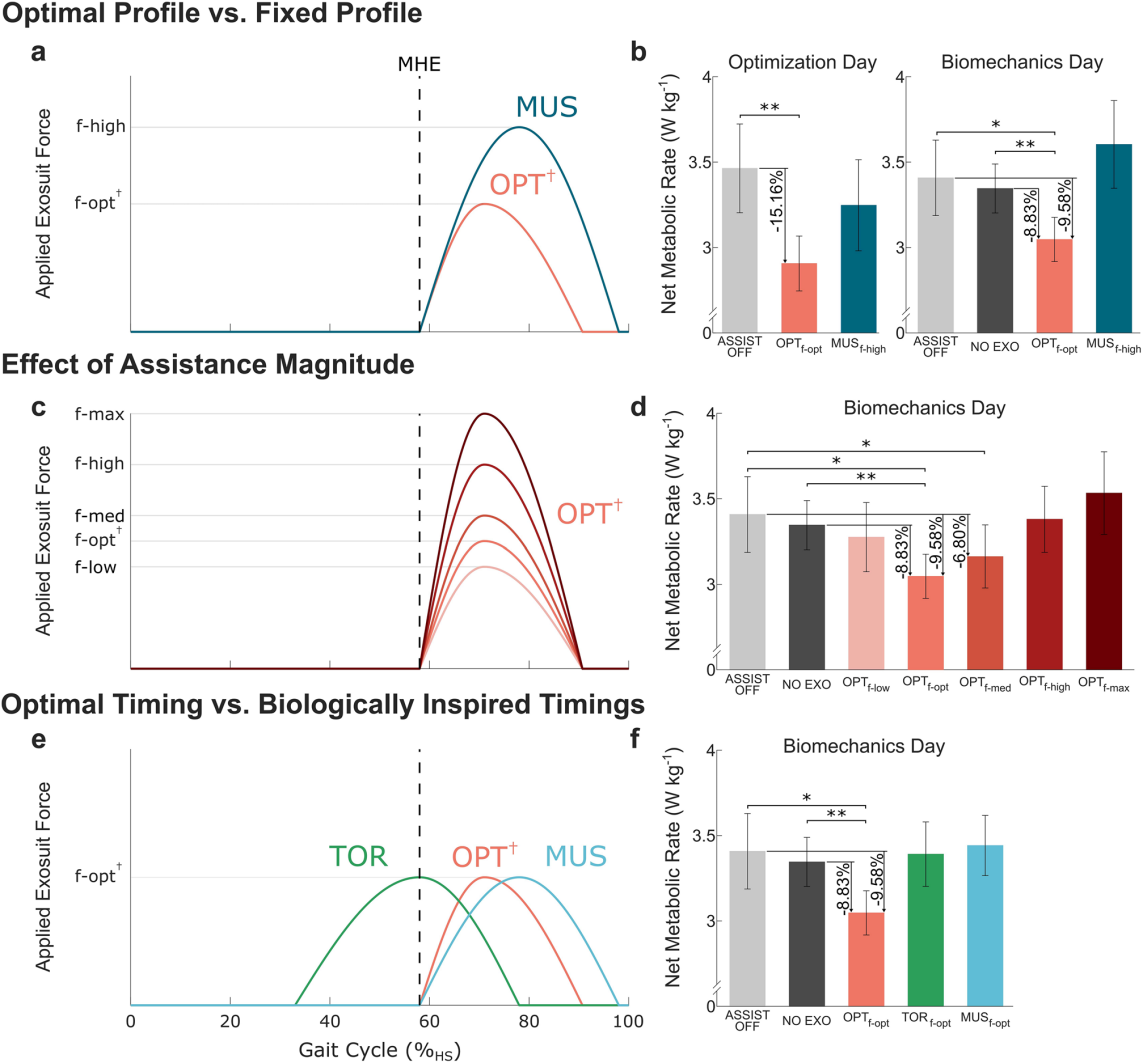
Metabolic results. (**a** and **b**) Comparison between the individualized optimal profile (${OPT}_{f-opt}$) identified on Optimization Day and a fixed profile (${MUS}_{f-high}$) that remains the same for all participants. Force profiles (**a**) and corresponding net metabolic rates (**b**) on Optimization Day (left) and Biomechanics Day (right). (**c** and **d**) Effect of assistance magnitude (${F}_{peak}$). Five different force magnitudes are evaluated: the optimal force ($f-opt$^†^), 60 N ($f-low$), 90 N ($f-med$), 120 N ($f-high$), and 150 N ($f-max$). The assistance timing for every profile is set to the optimal timing (*OPT*^†^). Force profiles (**c**) and corresponding net metabolic rates (**d**) on Biomechanics Day. (**e** and **f**) Effect of assistance timing. Three different assistance timings are evaluated: optimal timing (*OPT*^†^), biological torque inspired timing ($TOR$), and muscle activation inspired timing ($MUS$). The assistance magnitude of every profile is set to the optimal force ($f-opt$^†^). Force profiles (**e**) and corresponding net metabolic rates on Biomechanics Day (**f**). Error bars indicate the standard error of the mean (SEM). The vertical dashed lines represent the instant of MHE. † indicates an individualized parameter. Asterisks indicate statistically significant differences (n = 8, two-sided paired *t* tests; *P < 0.05; **P < 0.01).

We compared the optimized condition (${OPT}_{f-opt}$) and a fixed muscle inspired condition (${MUS}_{f-high}$) on both the optimization and biomechanics testing day (Fig. 3a and b) (Supplementary Methods). On the optimization day (Supplementary Video S1), the optimal profile resulted in a 15.2 ± 2.6% (0.56 ± 0.13 W kg^−1^; *P* < 0.01) reduction in net metabolic cost relative to the $ASSIST \: OFF$ (Supplementary Table S2). The estimated metabolic reduction relative to $NO \: EXO$ was 14.8 ± 2.7% (0.51 ± 0.13 W kg^−1^) based on the estimated metabolic cost of the 0.25 kg and 0.44 kg added to the participant’s waist and thigh respectively when wearing the textile components of soft exosuit (0.044 W kg^−1^)^54^. Subsequently, on the biomechanics day (Supplementary Video S2), the optimal profile resulted in a 9.6 ± 2.8% (0.36 ± 0.12 W kg^−1^; *P* < 0.05) reduction in net metabolic cost relative to the $ASSIST \: OFF$ and an 8.8 ± 1.7% (0.30 ± 0.06 W kg^−1^; *P* < 0.01) reduction relative to $NO \: EXO$ (Supplementary Table S3). The difference in net metabolic cost reduction between the optimization and biomechanics testing day could be attributed to exoskeleton assistance training and human motor adaptation^55^, a point expanded upon in the discussion. The fixed profile did not show a statistically significant change in metabolic cost relative to baseline on either the optimization or biomechanics day, providing further evidence for using optimization over a fixed profile. Additionally, on the biomechanics day, walking with the device unpowered ($ASSIST \: OFF$) increased the net metabolic cost by 1.4 ± 3.3% (0.06 ± 0.11 W kg^−1^; *P* = 0.59) relative to $NO \: EXO$. This measured cost of the added mass aligned well with the estimate of 0.044 W kg^−1^^54^.

### Effect of assistance magnitude

There was a quadratic relationship (*P* < 0.01) between peak exosuit torque magnitude ($\tau$; Nm kg^−1^) and net metabolic rate ($Met$; W kg^−1^) over the range of assistance magnitudes tested with minimum located between a low and medium magnitude indicating that neither too low nor too high assistance was metabolically beneficial (Fig. 3c and d, Supplementary Table S4) ($Met=12.74{\tau }^{2}-2.79\tau +3.40$; Intercept: *P* < 0.001, Linear term: *P* = 0.06, Quadratic term: *P* < 0.05; ${\tau }_{min}$ = 0.11 Nm kg^−1^). The only force magnitude that had a significant metabolic reduction was the optimal timing at a medium magnitude (${OPT}_{f-med}$) which demonstrated a 6.8 ± 2.7% (−0.25 ± 0.10 W kg^−1^; *P* < 0.05) reduction relative to $ASSIST \: OFF$.

**Figure 4:**
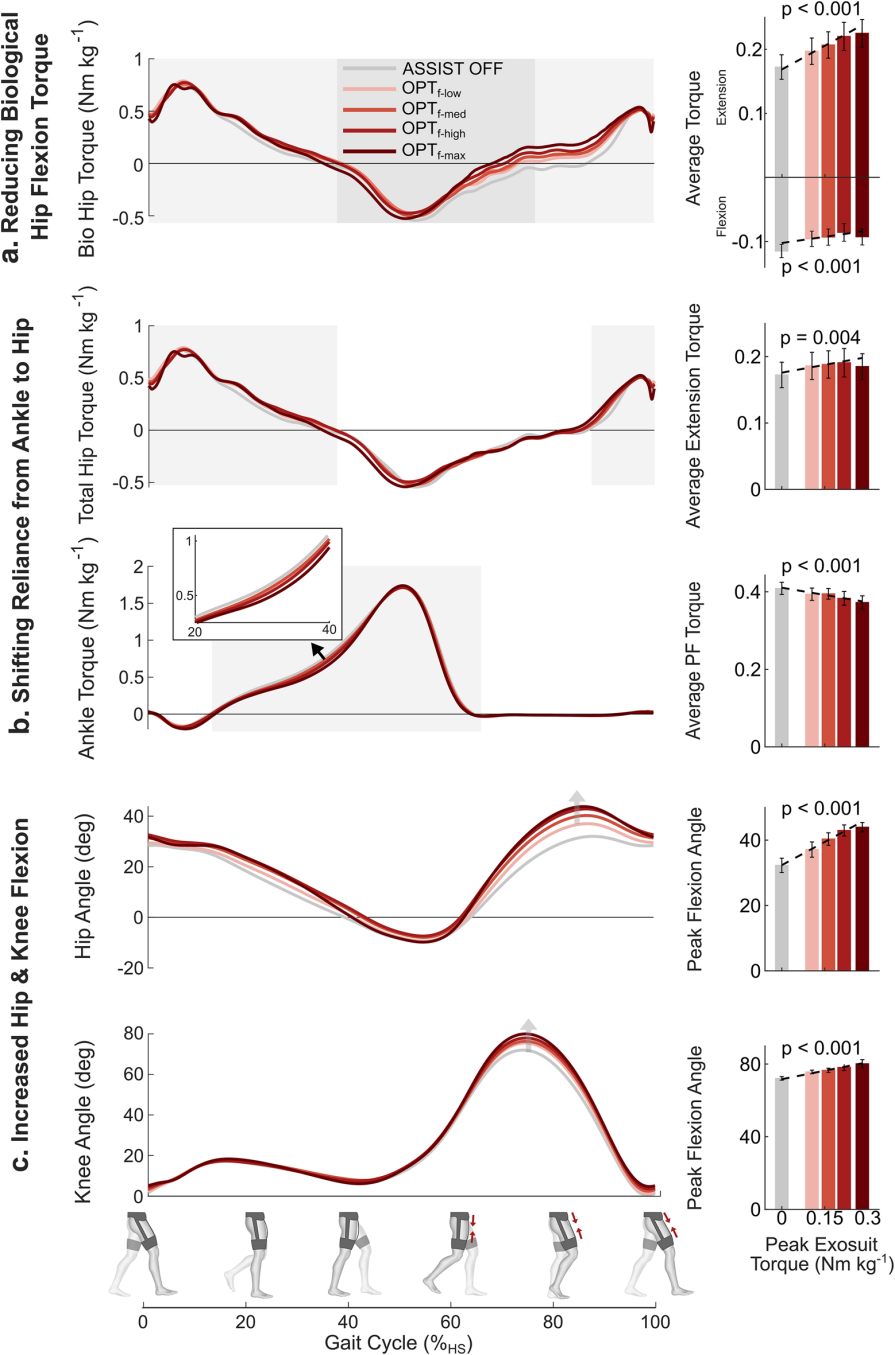
Biomechanics effects of assistance magnitude. Comparison between $ASSIST \: OFF$ and optimal timing at four different assistance magnitudes (${OPT}_{f-low}$, ${OPT}_{f-med}$, ${OPT}_{f-high}$, and ${OPT}_{f-max}$). (**a**) (left) Biological hip torque over the gait cycle. (right) Average biological hip extension torque which was found to increase with increasing assistance magnitude ($y=0.24\tau +0.17$; R^2^ = 0.98; *P* < 0.001) and average biological hip flexion torque which was found to decrease with increasing assistance magnitude ($y=0.08\tau -0.10$; R^2^ = 0.98; *P* < 0.001) (**b**) (left) Total hip extension and ankle plantarflexion torque over gait cycle. (right) Average total hip extension torque increased ($y=0.09\tau +0.18$; R^2^ = 0.98; *P* < 0.01) and average ankle plantarflexion torque decreased ($y=-0.15\tau +0.42$; R^2^ = 0.95; *P* < 0.001) with increasing assistance magnitude. (**c**) (left) Hip and knee joint angles across gait cycle. (right) Both peak hip flexion ($y=53\tau +32$; R^2^ = 0.82; *P* < 0.001) and peak knee flexion ($y=34\tau$+72; R^2^ = 0.72; *P* < 0.001) increased with increasing assistance magnitude. Shaded regions indicate areas of analysis. Error bars indicate the standard error of the mean (SEM). Equations are the result of a random intercept mixed-effects model with a linear regression and *P* values represent the statistical significance of the equation slope.

**Figure 5:**
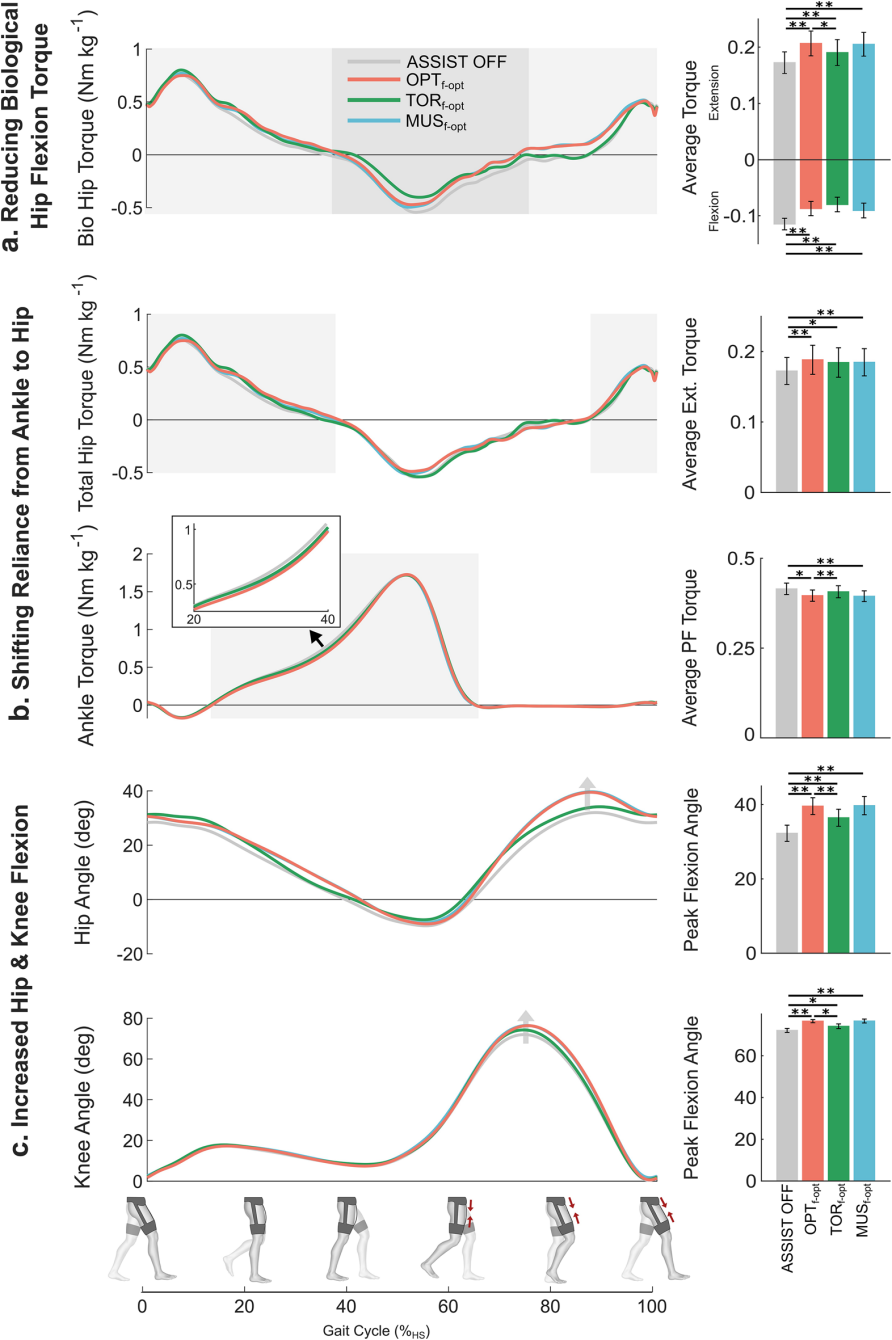
Biomechanics effects of assistance timing. Comparison between $ASSIST \: OFF$ and three different actuation timings at the same assistance magnitude ($f-opt$). Three conditions were individualized timing resulting from HIL (${OPT}_{f-opt}$), biological torque inspired timing (${TOR}_{f-opt}$) and biological muscle inspired timing (${MUS}_{f-opt}$). (**a**) (left) Biological hip torque over the gait cycle. (right) Average biological hip flexion and extension torque. (**b**) (left) Total hip extension and ankle plantarflexion torque over the gait cycle. (right) Average total hip extension torque and average ankle plantarflexion torque. (**c**) (left) Hip and knee joint angles across the gait cycle. (right) Peak hip flexion and peak knee flexion angle. Shaded regions indicate areas of analysis. Error bars indicate the standard error of the mean (SEM). Asterisks indicate statistically significant differences (n = 8, two-sided paired *t* tests; *P < 0.05; **P < 0.01).

In order to better understand how the exosuit assistance was enabling these metabolic reductions, we investigated how the kinetics and kinematics of the hip, knee, and ankle changed with increasing assistance magnitude. To isolate the active biomechanical effect of assistance from the passive effects of wearing the suits, we compared all biomechanical changes in the active conditions with respect to $ASSIST \: OFF$ (Figs. 4 and 5). Increasing assistance magnitude resulted in both positive biomechanical changes (i.e., reduced biological torques) and negative biomechanical changes (i.e., exaggerated hip and knee flexion), which explains why neither too low nor too high assistance was metabolically beneficial. With increasing assistance magnitude, average biological hip flexion torque magnitude (R^2^ = 0.98; *P* < 0.001) decreased and average biological hip extension torque magnitude (R^2^ = 0.98; *P* < 0.001) increased (Fig. 4a). Additionally, with increasing assistance magnitude there was a larger shift in total torque from ankle to hip (Fig. 4b) including a reduction in average total ankle plantarflexion torque (R^2^ = 0.95; *P* < 0.001) and an increase in average total hip extension torque (R^2^ = 0.98; *P* < 0.01). Finally, with the increase in assistance magnitude maximum hip flexion (R^2^ = 0.82; *P* < 0.001) and knee flexion (R^2^ = 0.72; *P* < 0.001) angles increased (Fig. 4c). A more detailed list of all the biomechanical parameters can be found in Supplementary Table S5.

### Optimal timing vs. biologically inspired timings

We compared three different actuation timings all with the same force magnitude (Fig. 3e and f). The optimal timing (${OPT}_{f-opt}$) was the only condition that showed a significant change in metabolic cost relative to $NO \: EXO$ or $ASSIST \: OFF$. The net metabolic rate of the optimal timing was lower than that of the torque (${TOR}_{f-opt}$) or muscle (${MUS}_{f-opt}$) inspired timing both on average and at every individual level (Supplementary Table S3). The optimal timing and the muscle inspired timing both delivered similar magnitudes of average positive (0.077 & 0.074 W kg^−1^) and negative (−0.008 & −0.009 W kg^−1^) exosuit power (Supplementary Table S4), but the torque inspired timing delivered significantly less average positive power (0.047 W kg^−1^) and significantly more negative power (−0.021 W kg^−1^) which could explain the little metabolic reduction.

The biomechanical changes of the three timings relative to $ASSIST \: OFF$ are summarized in Fig. 5. The optimal timing (${OPT}_{f-opt}$) demonstrated significant biomechanical changes relative to $ASSIST \: OFF$ including a decrease in average biological hip flexion torque and increase in average biological hip extension torque (Fig. 5a), shifting of average torque during midstance from ankle plantarflexion to hip extension (Fig. 5b), and increases in peak hip and knee flexion angles (Fig. 5c). Interestingly, the muscle inspired timing (${MUS}_{f-opt}$) resulted in very similar biomechanical changes including no significant differences with ${OPT}_{f-opt}$ in any of the metrics shown in Fig. 5, but these two timings had very different metabolic results. However, the torque inspired timing (${TOR}_{f-opt}$) did demonstrate biomechanical changes relative to the optimal timing including reduced average biological hip extension torque but still significant increases relative to $ASSIST \: OFF$ (Fig. 5a), increased average ankle plantarflexion torque with no significant change relative to $ASSIST \: OFF$ (Fig. 5b), and decreased peak hip and knee flexion angles although still significant increases relative to $ASSIST \: OFF$ (Fig. 5c). A more detailed list of how all the biomechanical parameters we investigated changed with different assistance timings can be found in Supplementary Table S6.

### Supplemental study (n = 3)—translating to an autonomous and portable exosuit

**Figure 6:**
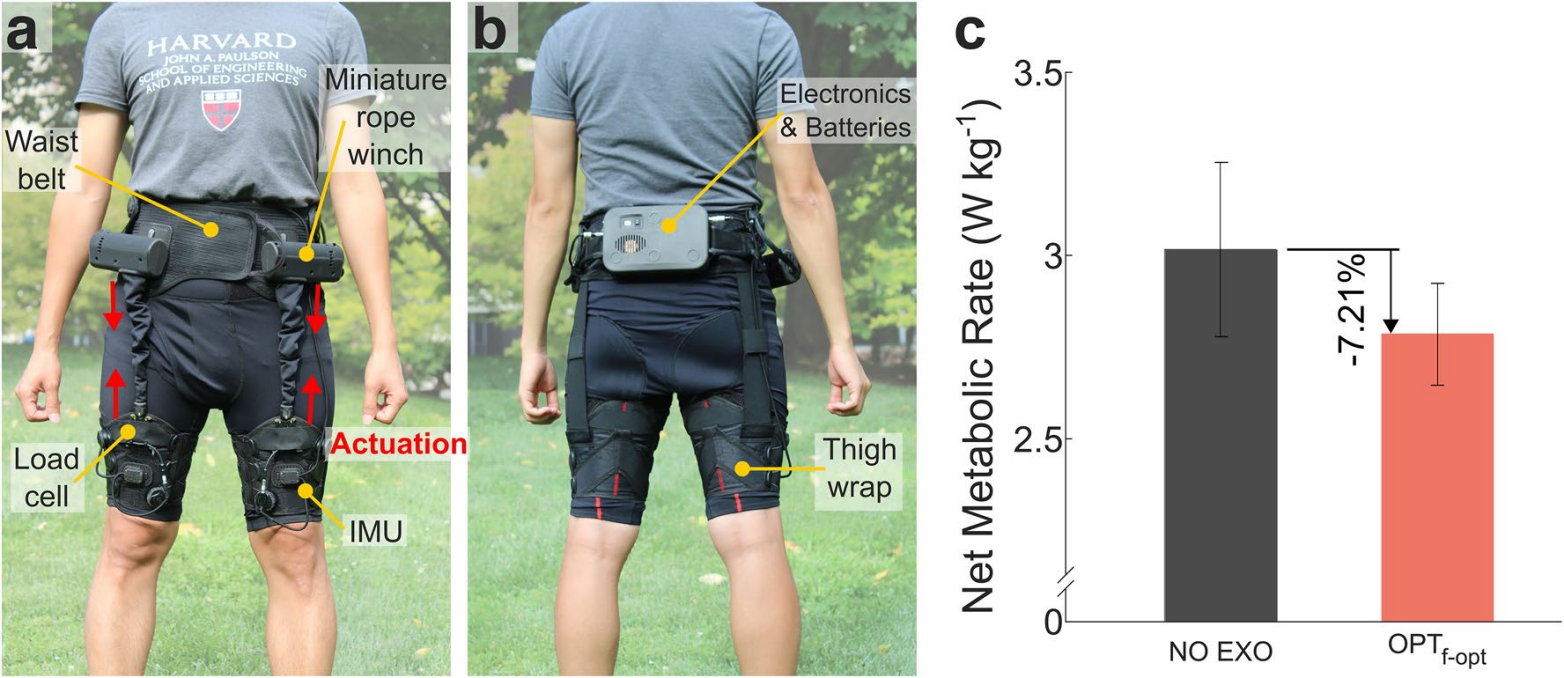
Autonomous hip flexion system & metabolic results. (**a** and **b**) Components of the autonomous and portable hip flexion exosuit. The front (**a**) and rear (**b**) view of the portable exosuit. (**c**) Metabolic results for portable exosuit with optimal timing (${OPT}_{f-opt}$) compared to normal walking ($NO \: EXO$). (n = 3; P2, P7, and P8 in Fig. 2 were recruited).

The low force and small power requirements identified in the optimal assistance profile are important aspects for a wearable assistive device to be used in a real-world, community setting: (i) a lightweight actuator minimizes the metabolic penalty to carry the additional weight of the device, (ii) a small form factor makes the device less obtrusive without restricting the user’s range of motion, and (iii) a small power usage makes the battery life last longer so that the device doesn’t require frequent charging throughout the day. To investigate the feasibility of autonomous and portable hip flexion assistance, we developed a portable hip flexion actuator based on the findings from the lab-based studies and evaluated its metabolic impact on a subset of three participants from the optimization protocol (Supplementary Video S3). This portable actuator was designed in a way that minimizes its size and weights while meeting the force and speed requirements to provide the optimal assistance profile. The total weight of the soft exosuit including actuator, batteries, sensors, and textile components was 2.31 kg (5.09 lbs), and it can be used for approximately 8 continuous hours of walking (Supplementary Methods). Applying participants’ optimal timings with a portable actuator resulted in a net metabolic reduction compared to $NO \: EXO$ of 7.2 ± 2.9% (0.23 ± 0.10 W kg^−1^) (Fig. 6 and Supplementary Table S7).

## Discussion

Individualized assistance of hip flexion resulted in a 15.2 ± 2.6% (0.56 ± 0.13 W kg^−1^) reduction in net metabolic cost relative to $ASSIST \: OFF$. This mean reduction in metabolic rate of 15.2% during walking (equivalent to 14.8% reduction relative to $NO \: EXO$) supports results from previous simulations studies highlighting that hip flexion may be a valuable aspect of gait to target with assistance. Relative to optimal assistance profiles found for other joint motions such as hip extension and ankle plantarflexion, the optimal hip flexion assistance profile identified here is both smaller in peak force/torque magnitude and more efficient at reducing metabolic cost (ratio of power input to metabolic reduction). This low force and high efficiency enable the possibility of a lightweight, low-profile mobile hip flexion exosuit that efficiently assists hip flexion.

The peak torque magnitude for the optimal assistance was only 0.146 Nm kg^−1^ or ~ 24% of peak biological hip flexion. Franks et al*.* used HIL optimization on a hip-only exoskeleton and found that across three participants the average optimal assistance magnitude on the hip extension was 0.404 Nm kg^−1^^47^, ~ 47% of peak biological hip extension torque (Supplementary Table S6; $NO \: EXO$ condition). Furthermore, Zhang et al*.* used HIL optimization to optimize ankle plantarflexion assistance and found that across eight participants the average optimal assistance magnitude was 0.76 Nm kg^−1^^8^, ~ 44% of peak biological ankle plantarflexion torque. For cable driven exoskeletons, the lower optimal torque magnitude for hip flexion also translates to a considerably lower force in the cable as the moment arms for hip flexion/extension and ankle plantarflexion are typically 15 cm^23^ and 10 cm^29^, respectively. Thus, the optimal peak torque and force for hip flexion was considerably lower than ankle plantarflexion and hip extension both in absolute magnitude and relative to the peak biological torque.

These low optimal peak force magnitudes enabled us to design a lightweight, portable exosuit which achieved a net metabolic reduction across three participants. The measured reduction with the autonomous system relative to $NO \: EXO$ (0.231 W kg^−1^; 7.2%) aligns well with the anticipated reduction (0.225 W kg^−1^; 6.6%) based on the ${OPT}_{f-opt}$ vs. $NO \: EXO$ results from the Biomechanics Day (0.298 W kg^−1^) and the estimated metabolic cost of the 1.62 kg added to the participant’s waist when wearing the autonomous system (0.073 W kg^−1^)^54^. The metabolic recording on this supplemental study occurred after participants walked for one hour with their previously identified optimal assistance, based on the estimates of the amount of time required to reach a steady-state minimum energy cost (ranges from 18 min^55^ to 90 min^56^) in the literature. However, the recent finding by Poggensee et al.^57^ showed that participants needed nearly 2 hours of exposure to active exoskeleton assistance to reach expertise and the amount of training time depended on the type of training experienced. Specifically, they showed that training with the low-variation in the assistance profile (as performed in our study) required twice longer training time to become an expert. Adaptation time could be highly context-dependent and the exposure time necessary to gain expertise in using an assistive device may vary widely. However, it could be possible to achieve higher metabolic reduction if we performed the longer training with moderate-variation in the assistance profile. Furthermore, we could achieve an even higher reduction by further optimizing the system beyond the current weight. The mean reduction in metabolic rate of 7.2% during walking is similar to the best-in-class reduction obtained with unpowered portable devices^40^ (7.2%) targeting independent hip flexion assistance. The individual metabolic reductions relative to $NO \: EXO$ for participants 2, 7, and 8 were 11.35% (0.38 W kg^−1^), 1.73% (0.04 W kg^−1^), and 8.54% (0.26 W kg^−1^), respectively. Although statistics were not run due to the small sample size, this feasibility study highlights the potential for translation to an autonomous and portable hip flexion exosuit.

The high metabolic reduction combined with the low optimal peak magnitude meant that the assistance provided was extremely efficient. Apparent joint efficiency^58^ (Supplementary Methods), the ratio of bilateral average positive exosuit power to the change in net metabolic rate relative to $ASSIST \: OFF$, was 0.28 on Optimization Day and 0.43 on Biomechanics Day for the optimal profile. The former of which is considerably lower than apparent efficiencies previously reported for hip extension, 0.50 (Supplementary Table S8) and ankle plantarflexion, 0.49 (Supplementary Table S9). Thus, on the Optimization Day, the ratio of metabolic reduction to the average positive exosuit power delivered was 1.8 times higher than ratios previously found for hip extension and ankle plantarflexion. These results align well with the apparent efficiencies of 0.2–0.4 measured in^34^ and suggest that under the right conditions the ratio of power input to metabolic reduction may be much more favorable for hip flexion than other joint motions.

The HIL optimization technique used in this work successfully individualized assistance profiles. The individualized, optimal profile, ${OPT}_{f-opt}$, varied considerably across participants, outperformed the fixed profile, and resulted in significant metabolic reductions relative to both $ASSIST \: OFF$ and $NO \: EXO$ conditions. The optimal profile suggested by HIL optimization showed the lowest metabolic rate on average among all eight different assist-on conditions tested. Furthermore, on an individual basis, the optimized assistance resulted in the best metabolic rate for three of the eight participants and was on average within 2.3 ± 0.4% of each individual’s best metabolic condition. As a one-dimensional partial validation of the optimal assistance magnitude, we performed a discrete step search of force magnitude across four fixed force levels tested. In the group level analysis, the medium force level (90 N) with a peak torque magnitude of 0.148 Nm kg^−1^, resulted in the largest metabolic reduction and the only significant reduction relative to $ASSIST \: OFF$ or $NO \: EXO$ besides the optimal force level (88.8 N). Furthermore, we were able to demonstrate that the average optimal force magnitude (88.8 N) aligned with the best performing force magnitude for each participant on average (88.1 N) (Supplementary Table S1).

Surprisingly, two biologically inspired control timings (i.e., muscle activation inspired profile and biological torque inspired profile), albeit prominent in the field, did not result in a significant metabolic reduction across participants, at the assistance levels explored in this study. Young et al*.* demonstrated a 13% reduction in net metabolic cost relative to $ASSIST \: OFF$ by using a muscle inspired timing, but they assisted both hip flexion and extension simultaneously so the portion of metabolic reduction solely from hip flexion assistance is not known^41^. In a very recent article, Zhou et al*.* used a torque inspired timing with a passive hip flexion exoskeleton and found a 7.2% reduction in net metabolic cost relative to $NO \: EXO$^40^. However, the peak exoskeleton torque delivered in Zhou’s study (0.099 Nm kg^−1^) was lower than the peak exosuit torque (0.141 Nm kg^−1^) in the torque inspired profile in this study. Zhou et al*.* also noted that they observed a net metabolic cost increase (+ 0.2%) when delivering higher peak exoskeleton torque (0.121 Nm kg^−1^) under the high spring stiffness conditions, which could explain why we did not see the significant metabolic reduction. In our work, we identified optimal assistance magnitude ($f-opt$) at the optimal assistance timing ($OPT$) and evaluated the effect of different actuation timings ($TOR$ and $MUS$) at that force magnitude ($f-opt$). However, it is possible that the biologically inspired control timings may perform better at lower assistance magnitudes.

On the Optimization Day (Day 2), the optimal assistance profile resulted in considerably larger metabolic reduction relative to $ASSIST \: OFF$ (−15.2%) than on Biomechanics Day (Day 3) (−9.6%). It is important to note there were several key differences between the Optimization Day and Biomechanics Day that may have influenced the metabolic results. The metabolic recording on Optimization Day occurred after participants walked for over two hours with exosuit assistance. The long duration walking may have induced fatigue or alternatively the exposure to the exosuit for this long period may have facilitated short-term learning that allowed participants to be more efficient during active assistance and/or less efficient during normal walking without exosuit. The Biomechanics Day had only thirteen minutes of warm-up (five minutes of $NO \: EXO$ and eight minutes of active assistance) before the metabolic conditions. In line with this finding, a recent study found that training required much more exposure than typical exoskeleton studies, about 109 min of assisted walking. Further, for their metabolic reductions, they found about half was attributed to training^57^.

To gain insights into the underlying cause of the metabolic reduction we analyzed the biomechanical changes of the optimal assistance relative to baseline. The optimal profile did display clear biomechanical benefits by reducing the average biological hip flexion torque and clear negative effects by inducing a marching type gait with increased hip and knee flexion during swing. Additionally, the optimal condition resulted in a reduction in average ankle plantarflexion torque and an increase in average total hip extension torque relative to $ASSIST \: OFF$. These changes occur predominantly during midstance when assistance is applied to the contralateral limb. This shift of reliance from the ankle to the hip somewhat aligns with the results found by Young et al.^34^ which showed reductions in peak ankle torque and peak positive ankle power along with increases in peak positive hip power across most of their active hip flexion conditions compared to walking with the exoskeleton powered off. However, those changes occurred predominantly during push-off whereas the changes in this work occurred predominantly during stance.

One challenge of analyzing the group level biomechanical results for optimization studies, particularly those with large ranging optimal settings, is that each individual is leveraging the assistance in a different way depending on their optimal timing and magnitude of assistance. For example, the average biomechanical results for the muscle inspired profile were very similar to the optimal profile but the two had very different metabolic results. Additionally, while the average optimal peak and offset timings were similar to the peak and offset timings of the muscle inspired profile (Supplementary Methods and Table S1), on an individual basis the optimal peak and offset timings deviated from the average by as much as 7.5% and 6.9% gait cycle, respectively. Thus, for some participants, the muscle inspired timing could peak/offset too early and for others, it could peak/offset too late but when performing group-level analysis those changes relative to the optimal timing averaged out.

There were a number of limitations in this study, including the fact that we only optimized over 3 parameters and held others constant such as onset timing and curvature of the profile. Due to fixed onset timing, the biological torque inspired profile (${TOR}_{f-opt}$) was outside the search space. Future studies which include a higher-dimensional parameter space including onset timing could lead to even further reductions. Additionally, our biomechanical comparison was conducted on a separate day than the optimization as we assumed that the optimal profile was applicable across days but given the findings in Supplementary Results and Discussions, this may not be the case. Finally, all participants in this study were males under the age of 50, in a future study it will be important to explore the benefits of hip flexion assistance for a more diverse population. For example, females are known to have a larger quadriceps angle, the angle formed between the quadriceps muscles and the patella tendon, which could affect the benefit gleaned from hip flexion assistance^59^. Furthermore, older adults may benefit differently from exosuit assistance due to the known physical and functional losses which occur during aging^30^.

## Conclusion

This study isolated the effects of independently assisting hip flexion during walking, a relatively unexplored joint motion, and showed that the resultant profile from HIL optimization resulted in a large metabolic reduction with surprisingly low levels of required assistance. The finding of the high ratio of metabolic reduction to exosuit power (i.e., low apparent joint efficiency) supports previous simulation findings^45,46^ and provides compelling evidence that hip flexion may be an efficient joint motion to target when considering how to create practical and lightweight wearable robots to support improved mobility. Additionally, the results of the study highlight the importance of individualized assistance with the optimal profile achieving significantly larger metabolic reductions both on average and for every individual as compared to both biological torque and muscle inspired assistive profiles which are prominent in the literature.

## Methods

The Harvard Longwood Medical Area Institutional Review Board (IRB) approved the studies, all experiments were performed in accordance with IRB approved guidelines and regulations, and all participants provided written informed consent. The participant included in the figures and videos provided written informed consent to publish the images/videos in an online open-access publication. Detailed materials and methods about soft exosuit, HIL optimization, and simulation of HIL optimization can be found in Supplementary Methods.

### Protocol

#### Main study

To identify the individualized optimal hip flexion profile and evaluate its effects on biomechanics and metabolic rate, we tested eight male participants (32.6 ± 7.5 years; 176.4 ± 6.6 cm; 73.8 ± 12.6 kg, SD) while walking at 1.25 m s^−1^ on a level treadmill. This protocol consists of three days of testing. Day 1 is a training day where the participants wear the suit active to get experience walking with assistance. Day 2 is an optimization day where we run HIL optimization in order to identify the participants’ optimal control timing parameters and force magnitude. After the optimization process was completed on Day 2, we measured the steady-state metabolic cost of walking with individualized optimal assistance (${OPT}_{f-opt}$), fixed assistance (${MUS}_{f-high}$), and walking with assistance turned off ($ASSIST \: OFF$). Day 3 is a biomechanics day where the participants walk with their individualized optimal assistance from Day 2 as well as several other assistance profiles for relative comparison of different biomechanics metrics. We measured the metabolic rate, kinematics and kinetics, and EMG for the $NO \: EXO$, $ASSIST \: OFF$, and assist-on conditions (${OPT}_{f-opt}$, ${OPT}_{f-low}$, ${OPT}_{f-med}$, ${OPT}_{f-high}$, ${OPT}_{f-max}$; ${MUS}_{f-opt}$, ${MUS}_{f-high}$; ${TOR}_{f-opt}$). The breaks between Day 1 and Day 2 as well as Day 2 and Day 3 were limited to between 4 and 8 days to minimize the time effects (e.g., the optimal profile change over time, training washout, etc.). More details about the experimental protocol can be found in the Supplementary Methods.

#### Supplemental study (n = 3)—translating to an autonomous and portable exosuit

To evaluate the feasibility of using a portable hip flexion system to reduce the metabolic cost of walking three participants (31.0 ± 4.4 years; 174.0 ± 9.5 cm; 67.3 ± 11.3 kg, SD) who already completed the optimization protocol were brought back for two additional days of testing. These tests were conducted approximately 11 months after the main three days protocol. On the first day, participants trained with a portable hip flexion exosuit set to their previously identified optimal control parameters for one hour while walking on the treadmill at 1.25 m s^−1^. On the second day, the metabolic impact of the portable actuator set to an individual's optimal parameter settings was evaluated using the Cosmed K5. After a five-minute standing metabolic trial and a one-hour active warm-up with the suit, active assistance using optimal parameters on a portable system (${OPT}_{f-opt}$) was evaluated relative to normal walking without a device ($NO \: EXO$). Both the ${OPT}_{f-opt}$ and $NO \: EXO$ conditions were repeated twice in a double-reversal order (ABBA). Each trial was 5 min in duration with 5 min of rest between trials. The net metabolic rates of the repeated conditions were averaged and the relative difference between the $NO \: EXO$ and ${OPT}_{f-opt}$ was calculated for each participant.

### Statistics

We organized the data and conducted statistical analyses in MATLAB (MathWorks, Natick, MA, USA). All results are reported as the mean ± standard error of the mean (SEM). When evaluating the relationship between the optimal peak torque magnitude and the body weight, we conducted a linear regression with a one-sample *t* test on the slope. The effects of the different conditions (multiple assist-on conditions, $ASSIST \: OFF$, and $NO \: EXO$) on metabolic rate were analyzed using the repeated measures ANOVA. Metabolic differences between conditions were evaluated with paired *t* tests. When evaluating the relationship between the net metabolic rate and assistance magnitude, we conducted a random intercept mixed-effects model with a quadratic function (random effect: participant-level intercept; fixed effect: linear and quadratic terms of peak exosuit hip flexion moment). To determine the order of polynomial function in the random intercept mixed-effects model, we used a likelihood ratio test comparing two nested models (e.g., linear versus quadratic relationship). When evaluating biomechanics effects of assistance magnitude, we conducted a random intercept mixed-effects model with a linear regression (random effect: participant-level intercept; fixed effect: the slope of peak exosuit hip flexion moment) (Fig. 4 and Supplementary Table S5). For biomechanical parameters related to the effect of the assistance timing, we compared the multiple assist-on conditions (${OPT}_{f-opt}$, ${TOR}_{f-opt}$, and ${MUS}_{f-opt}$) and the $ASSIST \: OFF$ condition using the repeated measures ANOVA. Differences in biomechanical parameters between conditions were evaluated with paired *t* tests (Fig. 5 and Supplementary Table S6).

## Supplementary Information

Supplementary Information 1.

Supplementary Video 1.

Supplementary Video 2.

Supplementary Video 3.

## Data Availability

All data needed to evaluate the conclusions of the paper are available in the paper or the Supplementary Information.
