# Supplementary material for: Reducing the energy cost of walking with low assistance levels through optimized hip flexion assistance from a soft exosuit
Source: Sci Rep. 2022 Jun 29;12:11004. doi: 10.1038/s41598-022-14784-9 (PMC9243082; doi:10.1038/s41598-022-14784-9)
Supplement: Supplementary file 1 — Supplementary Information 1. [file 41598_2022_14784_MOESM1_ESM.pdf]

Supplementary Information for

Reducing the energy cost of walking with low assistance levels through optimized hip flexion assistance from a soft exosuit

Jinsoo Kim[1,+], Brendan T. Quinlivan[1,+], Lou-Ana Deprey[1,2], Dheepak Arumukhom Revi[1,3,4], Asa Eckert-Erdheim[1], Patrick Murphy[1], Dorothy Orzel[1], and Conor J. Walsh[1,*]

[1]John A. Paulson School of Engineering and Applied Sciences, Harvard University, Boston, 02134, US
[2]School of Life Sciences, Swiss Federal Institute of Technology (EPFL), Lausanne, Switzerland
[3]Department of Mechanical Engineering, Boston University, Boston, 02215, US
[4]College of Health and Rehabilitation Sciences: Sargent college, Boston University, Boston, 02215, US
[*]walsh@seas.harvard.edu
[+]these authors contributed equally to this work

This PDF file includes:

Methods
Results
Discussion
References
Figures S1 to S3
Tables S1 to S9
Legends for Videos S1 to S3

Other supplementary materials for this manuscript include the following:

Videos S1 to S3

Methods

- **Soft Exosuit**
  - Textiles

    We designed the soft exosuit to assist hip flexion, which is slightly modified from a portable hip extension exosuit[1]. The functional apparel components of the exosuit consist of a spandex base layer (200 g), a waist belt (250 g), and two thigh wraps (2×146 g) (Fig. 1d). The base layer incorporates high-friction materials (NuStim, FabriFoam, USA) to help the exosuit remain attached to the wearer. We constructed the waist belt and thigh wraps with layers of an inextensible, abrasion-resistant plain-weave textile and a lightweight custom sailcloth material to evenly distribute the pressure around the iliac crests. The thigh wraps can be adjusted with laces and a tensioning dial (L4, Boa Technology, USA), and the waist belt can be tightened using a Velcro fastener. Detailed schematics of the waist belt, thigh wrap, and base layer construction can be found in the supplementary material of Kim *et al.*[1]. The total added mass of the system relative to $NO\ EXO$ condition in the main study was 0.69 kg of which 0.25 kg was concentrated on the waist (the mass of the waist belt) and 0.22 kg on each thigh (including the thigh brace, IMU, and load cells). The spandex base layer was worn in all conditions.

  - Off-board actuator and sensors

    We used a non-portable and tethered two-degrees-of-freedom (DoF) actuation system to provide the hip flexion assistance moment through Bowden cables. Anchoring points of the Bowden cable are at the bottom left and right of the waist belt and at the top center of the thigh wrap from an anterior view. On the actuator side, we attached the inner Bowden cable and its outer sheath to the pulley and pulley cover frame, respectively. On the exosuit side, we connected the inner Bowden cable and its outer sheath to the anchoring point of thigh wrap and waist belt using metal buckles (COBRA FM, AustriAlpin, Canada), respectively. When the actuator retracts, the distance between the two anchoring points on the waist belt and thigh wrap is shortened and tension is generated in the cable to assist hip flexion. Two load cells (2×43g) (LSB200, FUTEK, USA) are integrated into the anchoring point of thigh wrap to measure the cable force, and two IMUs (2×33g) (MTi-3 AHRS, Xsens, Netherlands) are secured by the plastic clip on the anterior part of thigh wrap to measure the thigh segment angle. More details of the actuator can be found in [2]. We used this off-board actuator for the main study and the repeating optimization experiment in the supplemental study.

  - Controls

    The electronics hardware has a three-layer configuration for the control system architecture: real-time target machine (top layer; Speedgoat, Switzerland), microprocessor (middle layer; ATSAME70N21, Atmel Co, USA), and a servo motor driver (bottom layer; Gold Twitter, Elmo Motion Control, Israel), and a separate, dedicated computer is used for the HIL optimization algorithm. This optimization computer collects the participant's metabolic data in real-time using an indirect calorimetry device (K5, Cosmed, Italy), runs the CMA-ES optimization algorithm in MATLAB (MathWorks, USA), and sends the assistance profile parameters that will be explored in the next condition to the real-time target machine. More details of the control system architecture can be found in [3].

    The high-level controller in a real-time target machine uses an IMU-based iterative algorithm that detects maximum hip extension (MHE) in every stride by identifying sign change in thigh angular velocity and estimates the wearer's gait cycle based on three

most recent stride times. This gait cycle is further divided into assisted and non-assisted phases. During the assisted phase, force control is used to create a hip flexion force profile with desired onset, peak, offset timings, and peak force magnitude. During the unassisted phase, position control is used to push out the cable in order not to disturb the wearer's limb motion and updates the cable position on a step-by-step basis to reach the desired pretension force just before switching to force control. While the high-level control is switching between force and position controllers, a low-level proportional-integral (PI) controller in Simulink (MathWorks, USA) with a servo motor driver in a torque control mode is used continuously. It is important to note that for two of the shorter participants, the short distance between the two anchoring points on the thigh wrap and waist belt limited the force tracking capabilities during the maximum force condition ($OPT_{f\text{-}max}$). During $OPT_{f\text{-}max}$ the assistance force was delivered with either lower peak force magnitude or earlier offset timing for 49.8% and 47.8% of the steps for participants 1 and 8, respectively.

- Portable system

  The portable system used a miniature rope winch design to generate the hip flexion torque. Two actuators were mounted onto the front of the waist using a waist belt and Dyneema rope spans the length of the thigh, attaching to the thigh wrap via a COBRA buckle (COBRA FM, AustriAlpin, Canada). Each winch contains an encoder (4096 cnt/rev; AS5145B, AMS, Austria) and one motor (U5, T-MOTOR, China) which transmits torque to a cylindrical drum (4.5 mm dia.) which is used to spool in Dyneema rope (1.8mm dia.; P/N KL0200, Marlow, USA). The electronics were mounted to the back of the waist belt and contained a custom-made electronics board using an Atmel processor (ATSAME70N21, Atmel Co, USA), motor driver (Gold Twitter, Elmo Motion Control Ltd, Israel), and two Li-Ion smart batteries (total 99.4Wh; RRC2054, RRC Power Solutions, Germany). Delivering the optimal assistance profile for a person with 80 kg body weight requires 12.3 W power usage, which enables approximately 8 continuous hours of walking with portable exosuit assistance. The same IMUs (MTi-3 AHRS, Xsens Technologies B.V., Netherlands), load cells (LSB200, FUTEK Advanced Sensor Technology, USA), and thigh wraps used in the main study were used as part of the portable system. The IMU-based iterative algorithm, the force and position switching controller, and the low-level PI controller were all implemented in standard C language (Visual Studio Code, Microsoft, USA). A waist belt (0.22 kg) similar in design to that used in the main study was used, with added attachments and adjustment for the miniature winch and electronics. The total weight of the system was 2.31 kg of which 1.87 kg was concentrated on the waist and 0.22 kg on each thigh. We used this portable system for the autonomous and portable exosuit experiment in the supplemental study.

- **Human-in-the-loop (HIL) Optimization**
  - Covariance matrix adaptation evolutionary strategy (CMA-ES)

    We used a Covariance Matrix Adaptation Evolutionary Strategy (CMA-ES) to identify three control parameters (T_{peak}, T_{offset}, and F_{peak}) (Fig. 1a) of the exosuit force profile that minimized the metabolic cost of walking for each participant[4,5]. The main objective of the covariance matrix adaptation is to fit the search distribution to the contour lines of an objective function to be minimized so that it can quickly approach the global optimum while minimizing the number of function evaluations. An overview of the algorithm is as follows.

CMA-ES first evaluates a group of candidate parameter sets that form the population of one generation. Candidate parameter sets are sampled from a multivariate normal distribution, characterized by a mean, a covariance matrix, and a standard deviation. The mean of the distribution represents the current estimate of the optimal parameter values, and the population size (λ) is determined based on the number of parameters (n) being optimized (e.g., λ = 7 for n = 3). After evaluating the current generation, the parameter sets are ranked in terms of performance and a rank-weighted average of the best sets (e.g., top three performers for n = 3) becomes the mean of the next generation. The locations of best parameter sets are used to update the covariance matrix of the next generation, while additional multi-generational correlation refines the covariance matrix and standard deviation. This process is repeated from generation to generation to imitate natural selection. Based on our simulation result, the eight generations were selected as a stopping criterion and a simple repair method[6] (i.e., if the sampled parameter set is outside of the boundary, this is moved to the closest feasible point) was chosen to handle boundary constraints. See Supplementary Results for more details on our simulation results. The mean of the final calculated generation serves as the best estimate of the optimal parameter. More details can be found in [7].

- Metabolic rate estimation

  The objective function of the CMA-ES algorithm is the steady-state metabolic rate of walking with hip flexion assistance. The steady-state metabolic rate corresponding to each exosuit control parameter set was estimated by fitting a first-order dynamics model[8,9] to two minutes of breath-by-breath transient-state metabolic measurements. We chose a sample duration of two minutes by considering the tradeoff between the estimation accuracy[7] of the first-order dynamics model and the total number of function evaluations given the limited time budget.

- **Protocol Details**
  - Day 1 (Training day)

    On the training day, we ran a CMA-ES optimization algorithm for four generations (about 1 hour) to expose the participant to a variety of different force profiles in the parameter space. Additionally, to allow the participant to experience the biological torque inspired profile (TOR) which is outside of parameter space, the participant walked with TOR profile at four different force magnitudes (f-low, f-med, f-$high$, f-max) for four minutes each.

  - Day 2 (Optimization day)

    On optimization day (Supplementary Video S1), we first measured the resting metabolic rate while participants stood still for 4 minutes. Then, the participants completed a 5-minute adaptation period of walking with a suit active at the starting condition of optimization. Participants then completed eight generations of HIL optimization (about 2 hours) with 5 minutes of rest and 5 minutes of adaptation in the middle. Each generation consisted of the objective function evaluation at seven different points in the parameter space, so there were 56 function evaluations in total (8 generations $\times$ 7 evaluation points/generation). The function evaluation was through 2 minutes of the metabolic rate estimation process. When transitioning from one profile to the other (i.e., between function evaluations), there were five transitioning strides where we gradually changed the assistance profile step by step to prevent a disturbance from an instantaneous change. After the optimization process was completed, we measured the metabolic cost of walking with individualized optimal assistance ($OPT_{f\text{-}opt}$), fixed assistance ($MUS_{f\text{-}high}$),

and walking with assistance turned off ($ASSIST\ OFF$). The $ASSIST\ OFF$ conditions were conducted at the beginning and repeated a second time at the end and the average was taken to avoid an order effect. Two assist-on conditions were randomized. Each condition lasted 5 minutes, and participants were allowed at least a 2-minute break between conditions.

- Day 3 (Biomechanics day)
  On biomechanics day (Supplementary Video S2), we measured the resting metabolic rate first. Then, the participants completed a five-minute warm-up of walking without a suit and an eight-minute warm-up with assistance. The eight minutes of active warm-up consisted of four different conditions, each two minutes in length. The four conditions were $TOR_{f\text{-}opt}$, one profile from MUS timing, and two profiles from OPT timing. The force levels for MUS and OPT timings are randomly chosen. Participants then completed a total of 11 conditions including two $NO\ EXO$, one $ASSIST\ OFF$, and eight different assist-on conditions. The two $NO\ EXO$ conditions were conducted at the beginning and end of the protocol and the average was taken to avoid an order effect. All eight of the assist-on conditions and the one $ASSIST\ OFF$ condition were randomized in the order. Each condition lasted 5 minutes, and participants were allowed at least a 2-minute break between conditions. We calculated the metabolic rate based on O_2 and CO_2 data gathered from the last two minutes of each condition using the Brockway equation[10].

  The eight assist-on conditions included three different assistance timings (OPT, TOR, MUS) and five different assistance magnitudes denoted as subscript ($f\text{-}opt$, $f\text{-}low$, $f\text{-}med$, $f\text{-}high$, and $f\text{-}max$). The eight conditions tested were: $OPT_{f\text{-}opt}$, $OPT_{f\text{-}low}$, $OPT_{f\text{-}med}$, $OPT_{f\text{-}high}$, $OPT_{f\text{-}max}$, $TOR_{f\text{-}opt}$, $MUS_{f\text{-}opt}$, and $MUS_{f\text{-}high}$.

  The three different timings were defined as follows. OPT was the individualized force profile timing resulting from HIL Optimization where the onset timing was fixed at MHE ($0\%_{MHE}$) and the peak timing and offset timing were individualized. TOR was inspired by biological hip flexion torque[11–14] with onset timing at $75\%_{MHE}$, peak timing at MHE ($0\%_{MHE}$), and offset timing at $20\%_{MHE}$. Finally, MUS was inspired by the electromyography (EMG) of hip muscles as shown in [13,15] with onset timing at MHE ($0\%_{MHE}$), peak timing at $20\%_{MHE}$, and offset timing at $40\%_{MHE}$.

  The five different force magnitudes included the individualized peak force magnitude resulting from HIL Optimization ($f\text{-}opt$) and constant peak force magnitudes corresponding to 60, 90, 120, and 150 N ($f\text{-}low$, $f\text{-}med$, $f\text{-}high$, and $f\text{-}max$, respectively).

- Supplemental study (n = 1) – Repeating optimization
  To evaluate if the individualized optimal profile remains constant over time, we repeated optimization day on one male participant (Participant 3; 29 years; 183cm; 93kg) 1 month after his first Optimization Day visit. We measured the resting metabolic rate, and then a participant completed eight generations of HIL optimization. After the optimization process was completed, we measured the metabolic cost of the $ASSIST\ OFF$ condition and two assist-on conditions: the old optimal assistance ($OPT^{old}_{f\text{-}opt}$) identified on Day 2 and the new optimal assistance ($OPT^{new}_{f\text{-}opt}$) found on re-optimization day. The $ASSIST\ OFF$ conditions were conducted at the beginning and repeated a second time at the end, and the metabolic rate was calculated as the average of the two. The old and new optimal assistance conditions were conducted in a double-reversal order. Each condition

was tested twice, first in random order, and then again in reverse order to mitigate the effects of adaptation or drift in respiratory measurements (order: old – new – new – old). The metabolic rate was calculated as the average of both double-reversal presentations.

- **Physiological and Biomechanical Measurements in the Main Study**
  - Metabolic measurements

    We used an indirect calorimetry device (K5, Cosmed, Italy) to measure O_2 consumption and CO_2 production. Participants were asked to refrain from alcohol, caffeine, and strenuous activities for twelve hours before the study and fast for two hours before the study. We calculated the metabolic rate based on O_2 and CO_2 data gathered from the last two minutes of each condition using the Brockway equation[10]. The net metabolic rate for each condition was obtained by subtracting the metabolic rate obtained during a standing trial performed at the beginning, from the metabolic power calculated during the walking conditions. All net metabolic rates and reductions were normalized to the participant's body mass.

  - Kinematics and kinetic measurements

    We measured body segment motions using a motion capture system (Qualisys, Sweden) and reflective markers placed according to a Helen-Hayes-type marker set. We measured ground reaction forces using an instrumented treadmill (Bertec, OH, USA) at 2 kHz. We filtered motion capture data and ground reaction force data using a zero-lag, fourth-order, low-pass Butterworth filter with a cutoff frequency at 7 Hz. We calculated joint kinematics and total (i.e., human plus the exosuit) joint moments and powers of the left leg in a sagittal plane using inverse kinematics and dynamics analyses in Visual3D (C-Motion, MD, USA). We normalized the total joint moments and powers by each participant's body mass. We calculated the exosuit joint moment by multiplying the forces from the exosuit load cell with a moment arm of the force path of the Bowden cable relative to the hip joint calculated using motion capture. We calculated the exosuit joint power by multiplying the exosuit moment by the hip joint angular velocity. We calculated the portion of the hip moment and power that is delivered by the biological muscles and passive tissues by subtracting the contribution of the exosuit from the total joint moment and power calculated by inverse dynamics. We used an automatic gait event detection algorithm (Visual3D, C-Motion, MD, USA) to determine heel strikes that defined gait cycles. Ten strides per condition were used for generating mean kinematic and kinetic data for each participant. The Qualisys data file which contained Motion Capture, GRF, and EMG data from the $OPT_{f\text{-}max}$ condition of one participant (Participant 3) was corrupted and thus was excluded from this analysis. Metabolic data which was logged separately and uncorrupted was still included.

  - Apparent joint efficiency

    To compute the apparent joint efficiency, we divided the average exosuit positive mechanical power (bilateral sum) by changes in net metabolic power as suggested in [16].

$$Apparent\ joint\ efficiency = \frac{Average\ exosuit\ positive\ mechanical\ power}{\Delta\ Net\ metabolic\ power} \quad (1)$$

The average exosuit positive mechanical power was calculated by integrating only the positive portions of the left exosuit mechanical power curves (from heel-strike to heel-strike), multiplying by 2 (with the assumption of symmetry between left and right legs), and dividing the total by the average stride period. The change in net metabolic power

was calculated by subtracting the net metabolic power during the optimal assistance condition ($OPT_{f\text{-}opt}$) from the net metabolic power during $ASSIST\ OFF$ condition.

- Electromyography measurements

  On Biomechanics Day, we measured activation of the left and right gluteus maximus, biceps femoris, rectus femoris, vastus lateralis, erector spinae and the left gastrocnemius medialis, soleus, and tibialis anterior muscles using surface electromyography (sEMG) at 2 kHz (Delsys, MA, USA). We processed sEMG data using a 20- to 450-Hz bandpass filter, rectification, and a 6-Hz low-pass filter. For each participant, we discarded parts of sEMG data based on visual inspection for artifacts due to sensor motion, sensor placement, sensor baseline drift, and cable actuation[17]. By default, we used sEMG data of the left leg for analysis, and we normalized sEMG based on the average of the two $NO\ EXO$ conditions. When artifacts were identified, we used sEMG data of the right leg (in 14.1% of all data), or we normalized sEMG data using only a single $NO\ EXO$ condition (in 15.5% of all data). Finally, if artifacts were identified in both legs we discarded the data for that condition (in 15.9% of data).

- **The Simulation of HIL Optimization**
  - Motivation

    One of the most important things in the HIL optimization study in the exoskeleton field is the experimental validation of the optimization algorithm (i.e., whether the optimization algorithm does truly find the optimum or not). Of the all HIL study on various lower-limb joints with different algorithms[3,7,8,18–20], only three studies experimentally validated their algorithms, and all were only one-dimensional parameter spaces[8,19,20]. This is mainly because the number of grid points that need to be sampled for experimental validation exponentially increases, as the dimension of parameter space increases. Furthermore, given the high noise level in the steady-state metabolic data, each point needs to be sampled multiple times to minimize the variability. Considering that a function evaluation (i.e., measuring the steady-state metabolic rate) at one point takes 5-6 minutes, it is extremely time-consuming and very difficult to experimentally prove that the HIL algorithm successfully found the global optimum, especially for higher-dimension optimization problems. Thus, we utilized simulation to confirm that this optimization procedure could find a global optimum under realistic metabolic noise conditions and to determine the best hyperparameter setting for the algorithm.

  - Parameter space

    Based on pilot experiments with hip flexion assistance, we found that both timing and magnitude of assistance cause a considerable change in the metabolic cost of assisted walking. Ideally, optimizing three timing parameters (onset, peak, and offset timings) and a peak force magnitude parameter is desirable. However, with further pilot testing, we found that the earlier onset timing is better, but onset timing earlier than MHE is detrimental to metabolic rate. Given that we had a limited time budget for four-dimensional optimization, we aimed to conduct three-dimensional optimization, where the three parameters were peak timing (T_{peak}), offset timing (T_{offset}), and the magnitude of peak force (F_{peak}) of the assistance profile (Fig. 1a). The onset timing was set to be $0\%_{\mathrm{MHE}}$. It is worthwhile to note that the biological torque inspired timing (TOR) was outside of parameter space but was included for the comparison for evaluation on Biomechanics Day.

We set a few constraints on these parameters based on hardware limitations and an underlying biomechanical rationale. The magnitude of peak force was set to be higher than 60 N and lower than 150 N. Forces lower than this range had a negligible impact on metabolic rate during pilot testing and force above this range had a clear negative impact on metabolic rate. The offset timing was set to be no later than $45\%_{\mathrm{MHE}}$ in order not to interrupt lower limb motion in an early stance phase. Finally, the duration between the onset and peak timings and the duration between the peak and offset timings were required to be at least 10% of the gait cycle due to the rising and falling time of the force controller.

- Objective function

The objective function of the optimization algorithm in our study is the metabolic rate of walking with hip flexion assistance, where the shape of the assistance profile is determined by three parameters (T_{peak}, T_{offset}, F_{peak}) to be optimized. To mimic the actual objective function in a simulation, we included additive zero-mean Gaussian noise to the noise-free ground-truth objective function. A ratio between the range of objective function and the amount of noise (i.e., signal variation to noise ratio) was set based on the literature and experimental pilot testing data. More details about this ratio can be found in the next section, signal variation to noise ratio (SNR). In this simulation, we assigned a ground-truth objective function as follows.

First, we assumed only one global optimum with no local optimum. The objective function with multiple local optima is a possible option, but the analysis will be complicated depending on the number of local optima, the relative locations of local optima with respect to global optimum, and the relative values of the objective function at the local optima with respect to that of the global optimum. Furthermore, there is no strong evidence that multiple local optima exist in the metabolic landscape in the literature. To draw a clear and simple conclusion, we assumed there was a single global optimum.

Second, we assumed that the objective function value increases linearly as the distance from the global optimum increases. Here, each parameter dimension is normalized to have a value between 0 and 1, and the Euclidean distance in the three-dimensional parameter space is used so that the distance is not dominated by one parameter due to their different units. This linear objective function is not an oversimplified assumption, because when projecting the linear objective function to lower-dimensional parameter space, the metabolic landscape will appear as a bowl-shape which is prevalent in many literatures.

Third, we did not fix the location of the global optimum in the parameter space across multiple simulation runs. Instead, we uniformly sampled 1000 points in the parameter space and used these points for each simulation run. The location of the global optimum point does affect the performance of the optimization algorithm. For example, the CMA-ES algorithm is initially designed for an unconstrained optimization problem, and thus it is vulnerable to the case where the ground-truth optimal point is located close to the boundary of the parameter space. Therefore, we systematically varied the location of the optimum point and reported the performance of the algorithm along with this optimum location change.

- Signal variation to Noise Ratio (SNR)

The ratio of the signal variation across the parameter space (i.e., the range of the objective function) relative to the noise in the measured signal has a considerable impact on the difficulty of the optimization problem (e.g., when SNR is small, finding the global optimum is extremely difficult). To estimate SNR, we identified the signal variation and the noise, respectively as follows.

To find the signal variation in the parameter space, we applied a variety of different assistance profiles to the participants and measured the net metabolic rate of walking during the pilot tests. The signal variation over the parameter space was found to be 1.223 W kg^{-1}.

Additionally, there are two different sources of metabolic noise: (i) the estimation error caused by instantaneous cost mapping and (ii) the inherent repeatability error in the metabolic data. The estimation error is caused by instantaneous cost mapping, the fitting process used to quickly estimate the steady-state metabolic cost for each iteration of the optimization process. At each iteration, we fit two minutes of transient data to the first-order dynamics of the human respiratory system with a known time constant[9] to estimate the steady-state metabolic rate for that condition. The estimation error would decrease if a longer duration of transient data is included in the fitting. However, with a fixed budget of total optimization time, there is a trade-off between minimizing estimation error and maximizing the number of iterations. When using the two minutes of transient data, the mean of estimation error was identified as 4.3% of the net metabolic rate[7]. We modeled this estimation error (e_{est}) as a zero-mean Gaussian noise, and the standard deviation (σ_{est}) of Gaussian noise can be identified as follows.

$$e_{est} \sim N(0, \sigma_{est}^2) \tag{2}$$

$$E[|e_{est}|] = \int_{-\infty}^{\infty} |x| \frac{1}{\sqrt{2\pi\sigma_{est}^2}} e^{-\frac{x^2}{2\sigma_{est}^2}} dx = \frac{2}{\sqrt{2\pi}} \sigma_{est} = 4.3\% \tag{3}$$

$$\sigma_{est} = 5.4\% \text{ of the net metabolic rate} \tag{4}$$

Even if we measure the steady-state metabolic data following standard practices (i.e., the participant performs the same activity for five minutes and the last two minutes of steady-state data is used to calculate the metabolic rate), there is an inherent repeatability error. This error can be easily seen by repeating the same condition multiple times and comparing the steady-state metabolic rate. The mean repeatability error varied from 2.6% to 4.8% of the net metabolic rate when analyzing the experimental data available in [21]. Similar to the estimation error, we modeled this repeatability error (e_{rep}) as a zero-mean Gaussian noise, and the standard deviation (σ_{rep}) of Gaussian noise can be identified as follows.

$$e_{rep} \sim N(0, \sigma_{rep}^2) \tag{5}$$

$$E\left[\left|e_{rep}\right|\right] = \int_{-\infty}^{\infty} |x| \frac{1}{\sqrt{2\pi\sigma_{rep}^2}} e^{-\frac{x^2}{2\sigma_{rep}^2}} dx = \frac{2}{\sqrt{2\pi}} \sigma_{rep} = 2.6 - 4.8\% \tag{6}$$

$$\sigma_{rep} = 3.3 - 6.0\% \text{ of the net metabolic rate} \tag{7}$$

There is quite large uncertainty in the repeatability error, so after doing the main three-day protocol, we validated the error range with experimental data in this study. The repeatability error was around σ_{rep} = 3.9% of the net metabolic rate in this study.

Finally, by assuming estimation and repeatability errors are independent, we can model the total error (e_{tot}) as a zero-mean Gaussian noise with the following standard deviation.

$$y = x + e_{est} + e_{rep} = x + e_{tot},\ e_{tot} \sim N(0, \sigma_{est}^2 + \sigma_{rep}^2) \quad (8)$$

$$\sigma_{tot} = \sqrt{\sigma_{est}^2 + \sigma_{rep}^2} = 6.7\% \text{ of the net metabolic rate} \quad (9)$$

where x means the noise-free ground-truth value of the net metabolic rate and y means the noisy measurement of the net metabolic rate.

When translating the standard deviation of the total error to signal variation (S), it was estimated as approximately 18.2%.

$$\sigma_{tot} \cong 0.182S \text{ (18.2\% of signal variation)} \quad (10)$$

- Performance metric
  To quantify the accuracy of the optimization algorithm, we used the normalized Euclidean distance between the ground-truth optimum and the point found by the algorithm. Again, we used normalized distance to avoid a biased result because of different parameter units.

  During pilot testing, it was difficult for the participant to differentiate the timing difference smaller than 3% or the force magnitude difference smaller than 15 N. Furthermore, we didn't see clear metabolic differences between two profiles whose timings and/or peak force differences are within 3% or 15 N. Based on these, we concluded that a good target for the optimization was to on average be within 3% of both timings and 15 N of the peak force, which is equivalent to a normalized distance of 0.238.

- Hyperparameters and simulation settings
  There are two important hyperparameters in CMA-ES to be determined through the simulation: (i) the number of generations (i.e., the total number of iterations) and (ii) the penalization method. Regarding the number of generations, if we run an optimization algorithm for only a small number of generations, the algorithm will not converge and fail to find the true optimum. On the other hand, if we run an algorithm with too many generations, we will increase the required walking time without improving the algorithm accuracy. Thus, we aimed to identify the appropriate number of generations by varying this hyperparameter between 1 and 10 in the simulation.

  The CMA-ES algorithm was originally devised for the unconstrained optimization problem but to use this algorithm in a constrained optimization problem, we need to penalize the objective function value when we sample the point outside of the parameter space. We explored three different penalization methods via simulation. The first method is a simple repair method[6] that moves an infeasible point to the closest feasible point in the parameter space (penalization method 1). The second method is resampling[6] any infeasible point until it becomes feasible (penalization method 2). The third method is evaluating the objective function on a repaired search point (i.e., closest feasible point) and adding a penalty depending on the distance to the repaired solution (penalization method 3). Here, a two-norm distance with an adaptive coefficient was used[22].

In addition to the two aforementioned hyperparameters, there are two key simulation settings: (i) the SNR and (ii) the location of the optimal point. Although we estimated the amount of noise as accurately as possible based on the literature and experimental data, there is still uncertainty in the SNR that is hard to be identified beforehand. Thus, we varied the SNR from 0% to 35% over 5% increments and evaluated how the algorithm accuracy changed as the noise level increased. Finally, since we don't have any information where the optimum point will be located in the parameter space, we uniformly sampled 1000 points from the parameter space and assigned each point as ground truth for one simulation run.

After running simulations for every possible combination of the aforementioned different hyperparameters and simulation settings, we calculated the mean and the standard error of the mean of the performance metric for 1000 different optimum locations to see the general trend with respect to the number of generations, the penalization method, and the SNR. In the simulations, we assigned a different seed number to the random number generator for different locations of ground truth optimum but kept the seed number the same when changing the other three simulation settings (the number of generations, the penalization method, and the SNR).

Results

- **The Simulation of HIL Optimization**
    - Covariance matrix adaptation evolutionary strategy (CMA-ES)

      The simulation result is shown in Supplementary Fig. S1. For all subfigures in Supplementary Fig. S1, the y-axis is the normalized distance between the ground-truth optimum and the optimal point found by the optimization algorithm. The smaller distance means improved algorithm accuracy. As we increase the number of generations, the algorithm accuracy is monotonically improved. However, this trend levels off after around the eighth generation (Supplementary Fig. S1a). Furthermore, among three different penalization methods tested, the simple repair method (moving an infeasible point to the closest feasible point) was the most accurate no matter how many generations were run. Here, the noise level was set to 20% of signal variation which is the closest option tested to the best estimate of the noise. Based on this result, we set hyperparameters of the CMA-ES algorithm as 8 generations and a simple repair method. Given the high uncertainty in the noise level, we plotted the performance metric over noise (Supplementary Fig. S1b) with these hyperparameter settings. When the noise level was 18.2% of signal variation which is the best estimate given the data available, the mean of normalized distance was 0.195, which equates to a 2.8% mean error in peak and offset timings and 10.1 N mean error in the peak force magnitude, which was better than our target of 0.238 mentioned above.

      As expected, the algorithm accuracy deteriorates as the noise level increases. Interestingly, this relationship (normalized distance between the ground truth and the algorithm solution versus standard deviation of zero-mean Gaussian noise) was close to linear (Supplementary Fig. S1b). When the ground-truth optimum is located closer to the boundary of the parameter space, the algorithm accuracy gets worse (Supplementary Fig. S1c and S1d). This trend is somewhat expected because if the optimum is closer to the boundary, there are higher chances that the infeasible points are sampled during the optimization process.

- Bayesian Optimization (BO)
  We repeated the exact same simulation setting with Bayesian optimization. After tuning hyperparameters (e.g., kernel function, kernel parameters, etc.) in BO, we compared the performance between BO and CMA-ES. Bayesian optimization was highly accurate and sample efficient, but it was less robust to the noise. Its performance quickly degraded when noise increased (e.g., Bayesian optimization was prone to suggest an incorrect optimal point located at the boundary.), and when the noise level reached 18.2% of signal variation which is the best estimate given the data available, Bayesian optimization performed worse than CMA-ES.

- **The Experiment of HIL Optimization**
  During human participant testing, we used the CMA-ES algorithm over eight generations with a simple repair method as boundary conditions to estimate optimal assistance profiles for each participant. Based on our preliminary simulation, the CMA-ES algorithm with these specific settings most accurately identified the optimal profile given the low signal-to-noise ratio (SNR) and the limited time budget for three-dimensional parameter space in this problem (Supplementary Results and Fig. S1).

  For each participant, the CMA-ES algorithm found the optimal assistance profile with a small scale of the search distribution at the last generation (Supplementary Fig. S2a). The objective function (i.e., metabolic rate) showed a decreasing trend as the algorithm iterated over generations and eventually leveled off at the end (Supplementary Fig. S2b), confirming that the optimization was run successfully with converged parameters (Supplementary Fig. S2c).

- **Supplemental Study (n = 1) – Repeating Optimization**
  As a supplemental study, we repeated the optimization on one participant as a preliminary investigation into how much the optimal settings changed over time and if the updated optimization settings resulted in a noticeably lower metabolic cost than the old settings. Thus, a month after the main study, we re-ran the HIL optimization on one participant to identify new optimal settings and then immediately experimentally measured the participant walking in those new settings ($OPT^{new}_{f\text{-}opt}$), the old optimal settings ($OPT^{old}_{f\text{-}opt}$) found during the main study above, and walking with no assistance ($ASSIST\ OFF$). We repeated each of the three conditions twice to account for the noise in the metabolic measurement. The results are shown in Supplementary Fig. S3. The old optimization settings were 20.6%$_{\mathrm{MHE}}$ peak, 39.9%$_{\mathrm{MHE}}$ offset, and 127 N; and resulted in a 6.9% (0.25 W kg^{-1}) metabolic reduction relative to $ASSIST\ OFF$. The new optimization settings were 12.4%$_{\mathrm{MHE}}$ peak, 33.2%$_{\mathrm{MHE}}$ offset, and 143 N; and resulted in an 8.6% (0.31 W kg^{-1}) metabolic reduction relative to $ASSIST\ OFF$. Thus, the optimal assistance settings did change (more than the minimum timing and peak force differences that can differentiate two profiles), and the new optimal profile performed metabolically better than the old optimal profile, although with only N=1, the metabolic difference between the two was not big enough to account for the repeatability error in the metabolic cost (Supplementary Methods).

- **Physiological and Biomechanical Measurements in the Main Study**
  - Kinematics and Dynamics
    To isolate the passive effects of wearing the suits from the active biomechanical effect of assistance, we compared $ASSIST\ OFF$ and $NO\ EXO$ conditions using paired t tests. It is worth noting that there were a few small but significant differences between the two, summarized in Supplementary Table S6.

  - Electromyography measurements

There were no significant changes in any of the sEMG signals between $ASSIST\ OFF$ and $NO\ EXO$. For $OPT_{f\text{-}opt}$, the integral of vastus lateralis during stance increased (0.059 $\pm$ 0.015; $P < 0.01$) relative to $ASSIST\ OFF$. There were no significant changes between $OPT_{f\text{-}opt}$ and $TOR_{f\text{-}opt}$. For $MUS_{f\text{-}opt}$, the integral of vastus lateralis during stance increased (0.064 $\pm$ 0.017; $P < 0.01$) relative to $ASSIST\ OFF$ but was not significantly different from $OPT_{f\text{-}opt}$. Finally increases in peak exosuit torque across the force sweep conditions ($OPT_{f\text{-}low}$, $OPT_{f\text{-}med}$, $OPT_{f\text{-}high}$, and $OPT_{f\text{-}max}$) resulted in a linear increase in the integral of tibialis anterior ($R^2 = 0.88$; $P < 0.001$), biceps femoris ($R^2 = 0.96$; $P < 0.01$), and erector spinae ($R^2 = 0.92$; $P < 0.01$) using a linear random intercept mixed-effects model.

Discussion

- **The Experiment of HIL Optimization**
  In this study, we were not able to experimentally prove that the HIL algorithm successfully found the global optimum. Given the three-dimensional parameter space and low signal-to-noise ratio of metabolic measurements, it would be very difficult to experimentally validate but we do have confidence it converged to the most accurately identified global optimum based on our preliminary simulation and CMA-ES algorithm experiment results (Supplementary Results).

- **Supplemental Study (n = 1) – Repeating Optimization**
  The optimal parameters utilized for the Optimization Day were a result of the HIL optimization run on that day but on the Biomechanics Day no optimization was run. Instead, we utilized the previously identified optimal parameters, assuming that those parameters still remained optimal across days. If these optimal parameters were to change between days, as found in the repeating optimization supplemental study (n = 1), that could also explain the smaller metabolic reduction found on the Biomechanics Day. However, there were no significant changes between Optimization Day and Biomechanics Day in the net metabolic rate of optimal assistance ($P = 0.41$), the net metabolic rate of $ASSIST\ OFF$ ($P = 0.87$), or the relative difference between optimal assistance and $ASSIST\ OFF$ ($P = 0.35$).

  The repeated optimization results indicate that the optimal profile may vary over time and thus optimizing an individual's profile periodically may be required. However, this is solely based on a single-participant test and further testing and validation of statistical significance are needed. If the optimal profile does vary over time, measuring indirect calorimetry is too time-intensive to implement in a real-world environment, and thus a different approach is needed if we want to optimize exoskeletons in daily life. Finding a proxy objective function that highly correlates with metabolic energy and that can be measured easily in real-time will be the first step of the new paradigm of optimization. Ingraham *et al.*[23] has initiated this work by systematically evaluating how multiple physiological signals (e.g., limb accelerations, lower limb EMG, heart rate, skin temperature), both alone and in combination, can predict the metabolic cost of various activities. They have used such physiological signals in tandem with metabolic measurements to increase the speed of predicting instantaneous metabolic cost[24] but have yet to develop a proxy function that does not use metabolic measurements. Further work is needed to identify an appropriate proxy function.

- **Physiological and Biomechanical Measurements in the Main Study**
  - Increasing average biological hip extension torque

During portions of the swing phase, exosuit torque and power exceeded that of net total hip torque and power. This resulted in increased average biological hip extension torque and average negative hip biological power in $OPT_{f\text{-}opt}$ and $MUS_{f\text{-}opt}$, and they were correlated with increases in peak exosuit torque during the force sweep conditions. The average biological hip extension torque increased between $ASSIST\ OFF$ and $OPT_{f\text{-}opt}$ by 0.032 $\pm$ 0.011 Nm kg^{-1} ($P < 0.001$) and between $ASSIST\ OFF$ and $MUS_{f\text{-}opt}$ by 0.029 $\pm$ 0.009 Nm kg^{-1} ($P < 0.001$). The average negative hip biological power increased in magnitude between $ASSIST\ OFF$ and $OPT_{f\text{-}opt}$ by -0.013 $\pm$ 0.011 W kg^{-1} ($P < 0.05$) and between $ASSIST\ OFF$ and $MUS_{f\text{-}opt}$ by -0.018 $\pm$ 0.012 W kg^{-1} ($P < 0.01$).

Due to the more net biological torque inspired timing there is lower average biological hip extension torque (-0.014 $\pm$ 0.012 Nm kg^{-1}; $P < 0.05$) and average negative hip biological power (0.026 $\pm$ 0.013 W kg^{-1}; $P < 0.001$) in $TOR_{f\text{-}opt}$ condition compared to $OPT_{f\text{-}opt}$. Although the average biological hip extension torque did still increase in magnitude relative to $ASSIST\ OFF$ (0.018 $\pm$ 0.014 Nm kg^{-1}; $P < 0.05$).

The increases in biological torque/power are not necessarily detrimental, they may just be revealing the natural underlying co-contraction of hip flexion and extension muscles during swing[25]. These findings align with the simulation results of Dembia *et al.* which found that when minimizing muscle actuation with hip flexion assistance, the device torque exceeded the net joint moment[26]. However, it is possible that during certain periods of the gait cycle the exosuit torques during the higher assistance conditions (120 and 150 N) exceeded those of the underlying musculature and negatively influenced metabolic cost.

- Electromyography measurements

  It is interesting that we don't see any significant reductions in EMG despite the fact that we see decreases in metabolic cost. Due to the constraints of surface EMG, the rectus femoris was the only muscle we measured which contributes to hip flexion. We were not able to measure anything from the four other muscles which contribute to hip flexion (iliacus, psoas, iliocapsularis, and sartorius). No significant changes in the peak or integral of the rectus femoris signal were found across conditions. The increases in tibialis anterior, biceps femoris, and erector spinae during force sweep are small but significant.

References

1. Kim, J. *et al.* Reducing the metabolic rate of walking and running with a versatile, portable exosuit. *Science* **365**, 668-672 (2019).
2. Lee, G. *et al.* Improved assistive profile tracking of soft exosuits for walking and jogging with off-board actuation. In *IEEE/RSJ International Conference on Intelligent Robots and Systems* 1699-1706 (2017).
3. Kim, M. *et al.* Bayesian optimization of soft exosuits using a metabolic estimator stopping process. In *IEEE Int. Conf. Robot. Autom.* 9173-9179 (2019).
4. Hansen, N. & Ostermeier, A. Completely derandomized self-adaptation in evolution strategies. *Evol. Comput.* **9**, 159-195 (2001).
5. Hansen, N. The CMA evolution strategy: a comparing review in *Towards a new evolutionary computation. Studies in fuzziness and soft computing.* **192**, 75-102 (Lozano, Larranaga, Inza & Bengoetxea, 2006).

6. Hansen, N. The CMA evolution strategy: A tutorial. Preprint at https://arxiv.org/abs/1604.00772 (2016).
7. Zhang, J. *et al.* Human-in-the-loop optimization of exoskeleton assistance during walking. *Science* **356**, 1280-1284 (2017).
8. Felt, W., Selinger, J. C., Donelan, J. M. & Remy, C. D. "Body-in-the-loop": Optimizing device parameters using measures of instantaneous energetic cost. *PLoS One* **10**, e0135342 (2015).
9. Selinger, J. C. & Donelan, J. M. Estimating instantaneous energetic cost during non-steady-state gait. *J. Appl. Physiol.* **117**, 1406-1415 (2014).
10. Brockway, J. M. Derivation of formulae used to calculate energy expenditure in man. *Hum. Nutr. Clin. Nutr.* **41**, 463-471 (1987).
11. Seo, K., Lee, J., Lee, Y., Ha, T. & Shim, Y. Fully autonomous hip exoskeleton saves metabolic cost of walking. In *IEEE Int. Conf. Robot. Autom.* 4628-4635 (2016).
12. Lewis, C. L. & Ferris, D. P. Invariant hip moment pattern while walking with a robotic hip exoskeleton. *J. Biomech.* **44**, 789-793 (2011).
13. Young, A. J., Gannon, H. & Ferris, D. P. A biomechanical comparison of proportional electromyography control to biological torque control using a powered hip exoskeleton. *Front. Bioeng. Biotechnol.* **5**, 37 (2017).
14. Lenzi, T., Carrozza, M. C. & Agrawal, S. K. Powered hip exoskeletons can reduce the user's hip and ankle muscle activations during walking. *IEEE Trans. Neural Syst. Rehabil. Eng.* **21**, 938-948 (2013).
15. Hsu, H., Kang, I. & Young, A. J. Design and evaluation of a proportional myoelectric controller for hip exoskeletons during walking. In *Proc. ASME Dyn. Syst. Control Conf.* (2018).
16. Sawicki, G. S. & Ferris, D. P. Mechanics and energetics of level walking with powered ankle exoskeletons. *J. Exp. Biol.* **211**, 1402-1413 (2008).
17. Jackson, R. W. & Collins, S. H. An experimental comparison of the relative benefits of work and torque assistance in ankle exoskeletons. *J. Appl. Physiol.* **119**, 541-557 (2015).
18. Ding, Y., Kim, M., Kuindersma, S. & Walsh, C. J. Human-in-the-loop optimization of hip assistance with a soft exosuit during walking. *Sci. Robot.* **3**, eaar5438 (2018).
19. Koller, J. R., Gates, D. H., Ferris, D. P. & Remy, C. D. 'Body-in-the-loop' optimization of assistive robotic devices: A validation study. In *Robot. Sci. Syst.* (2016).
20. Kim, M. *et al.* Human-in-the-loop bayesian optimization of wearable device parameters. *PLoS One* **12**, e0184054 (2017).
21. Quinlivan, B. T. *et al.* Assistance magnitude versus metabolic cost reductions for a tethered multiarticular soft exosuit. *Sci. Robot.* **2**, eaah4416 (2017).
22. Hansen, N., Niederberger, A. S. P., Guzzella, L. & Koumoutsakos, P. A method for handling uncertainty in evolutionary optimization with an application to feedback control of combustion. *IEEE Trans. Evol. Comput.* **13**, 180-197 (2009).
23. Ingraham, K. A., Ferris, D. P. & Remy, C. D. Evaluating physiological signal salience for estimating metabolic energy cost from wearable sensors. *J. Appl. Physiol.* **126**, 717-729 (2019).
24. Ingraham, K. A., Rouse, E. J. & Remy, C. D. Accelerating the estimation of metabolic cost using signal derivatives: Implications for optimization and evaluation of wearable robots. *IEEE Robot. Autom. Mag.* **27**, 32-42 (2020).
25. Strazza, A. *et al.* Surface-EMG analysis for the quantification of thigh muscle dynamic co-contractions during normal gait. *Gait Posture* **51**, 228-233 (2017).
26. Dembia, C. L., Silder, A., Uchida, T. K., Hicks, J. L. & Delp, S. L. Simulating ideal assistive devices to reduce the metabolic cost of walking with heavy loads. *PLoS One* **12**, e0180320 (2017).
27. Ding, Y. *et al.* Effect of timing of hip extension assistance during loaded walking with a soft exosuit. *J. Neuroeng. Rehabil.* **13**, 87 (2016).
28. Ding, Y. *et al.* Biomechanical and physiological evaluation of multi-joint assistance with soft exosuits. *IEEE Trans. Neural Syst. Rehabil. Eng.* **25**, 119-130 (2017).

29. Panizzolo, F. A. *et al.* Metabolic cost adaptations during training with a soft exosuit assisting the hip joint. *Sci. Rep.* **9**, 9779 (2019).
30. Sawicki, G. S. & Ferris, D. P. Powered ankle exoskeletons reveal the metabolic cost of plantar flexor mechanical work during walking with longer steps at constant step frequency. *J. Exp. Biol.* **212**, 21-31 (2009).
31. Malcolm, P., Derave, W., Galle, S. & De Clercq, D. A simple exoskeleton that assists plantarflexion can reduce the metabolic cost of human walking. *PLoS One* **8**, e56137 (2013).
32. Mooney, L. M., Rouse, E. J. & Herr, H. M. Autonomous exoskeleton reduces metabolic cost of human walking. *J. Neuroeng. Rehabil.* **11**, 151 (2014).
33. Koller, J. R., Jacobs, D. A., Ferris, D. P. & Remy, C. D. Learning to walk with an adaptive gain proportional myoelectric controller for a robotic ankle exoskeleton. *J. Neuroeng. Rehabil.* **12**, 97 (2015).
34. Mooney, L. M. & Herr, H. M. Biomechanical walking mechanisms underlying the metabolic reduction caused by an autonomous exoskeleton. *J. Neuroeng. Rehabil.* **13**, 4 (2016).
35. Galle, S., Malcolm, P., Collins, S. H. & De Clercq, D. Reducing the metabolic cost of walking with an ankle exoskeleton: interaction between actuation timing and power. *J. Neuroeng. Rehabil.* **14**, 35 (2017).
36. Poggensee, K. L. & Collins, S. H. How adaptation, training, and customization contribute to benefits from exoskeleton assistance. *Sci. Robot.* **6**, eabf1078 (2021).
37. Franks, P. W. *et al.* Comparing optimized exoskeleton assistance of the hip, knee, and ankle in single and multi-joint configurations. *Wearable Technol.* **2** (2021).

Figures

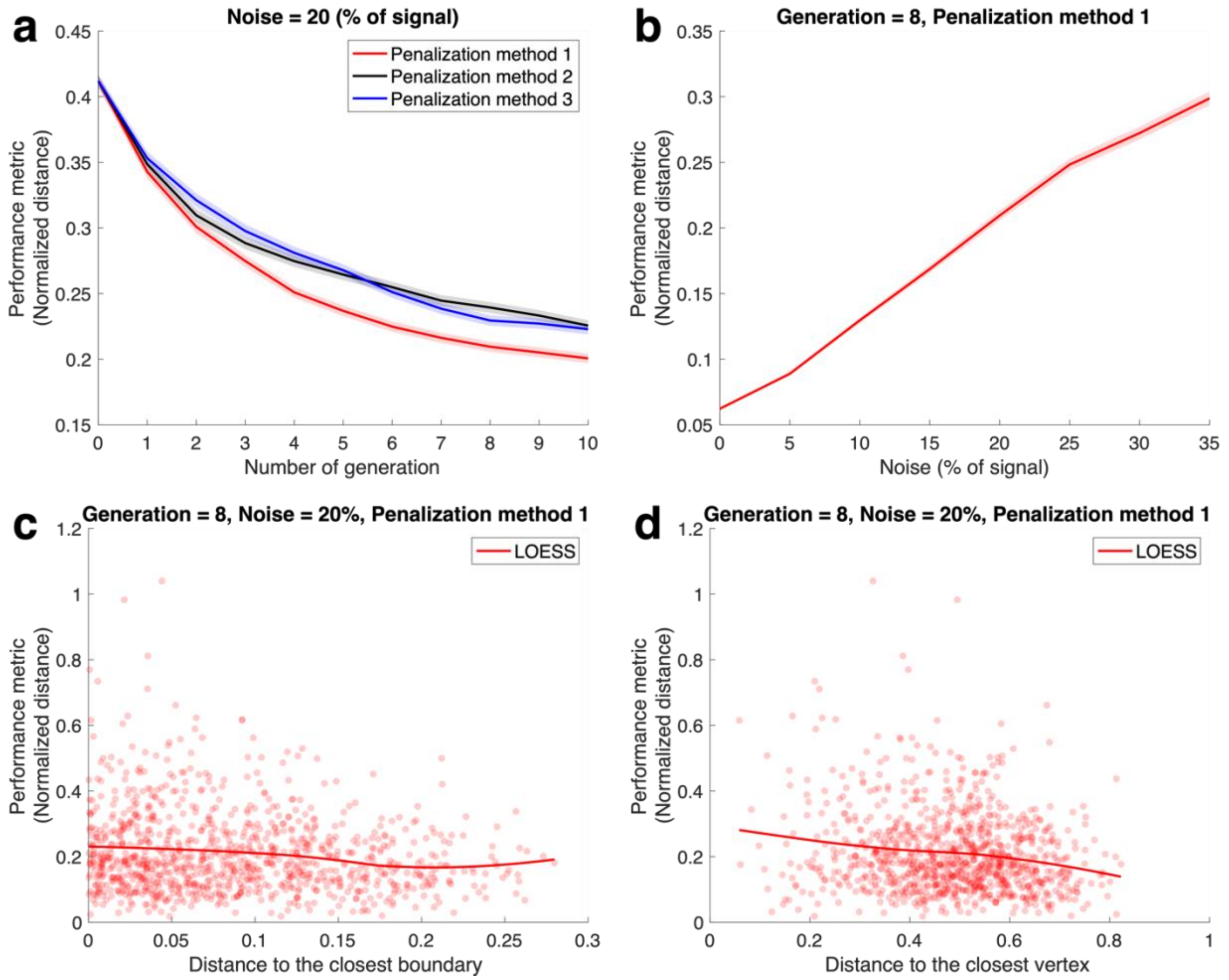

Figure S1. Simulation Result. The y-axes for all subfigures are the performance metric, the normalized Euclidean distance between the ground-truth optimum and the point found by the algorithm. The solid lines and the shaded regions represent the mean and the standard error of the mean, respectively. (**a**) The performance metric versus the number of generations for the three different penalization methods. Penalization method 1 is moving an infeasible point to the closest feasible point in the parameter space. Penalization method 2 is resampling an infeasible point until it becomes feasible. Penalization method 3 is adding a penalty based on the distance to the repaired solution. (**b**) The performance metric versus noise level. (**c**) The performance metric versus the distance from the ground-truth optimum to the closest boundary of the parameter space. The solid line is the fitting of a locally estimated scatterplot smoothing (LOESS) model (span = 0.75). (**d**) The performance metric versus the distance from the ground-truth optimum to the closest vertex of the parameter space. The solid line is the LOESS model (span = 0.75).

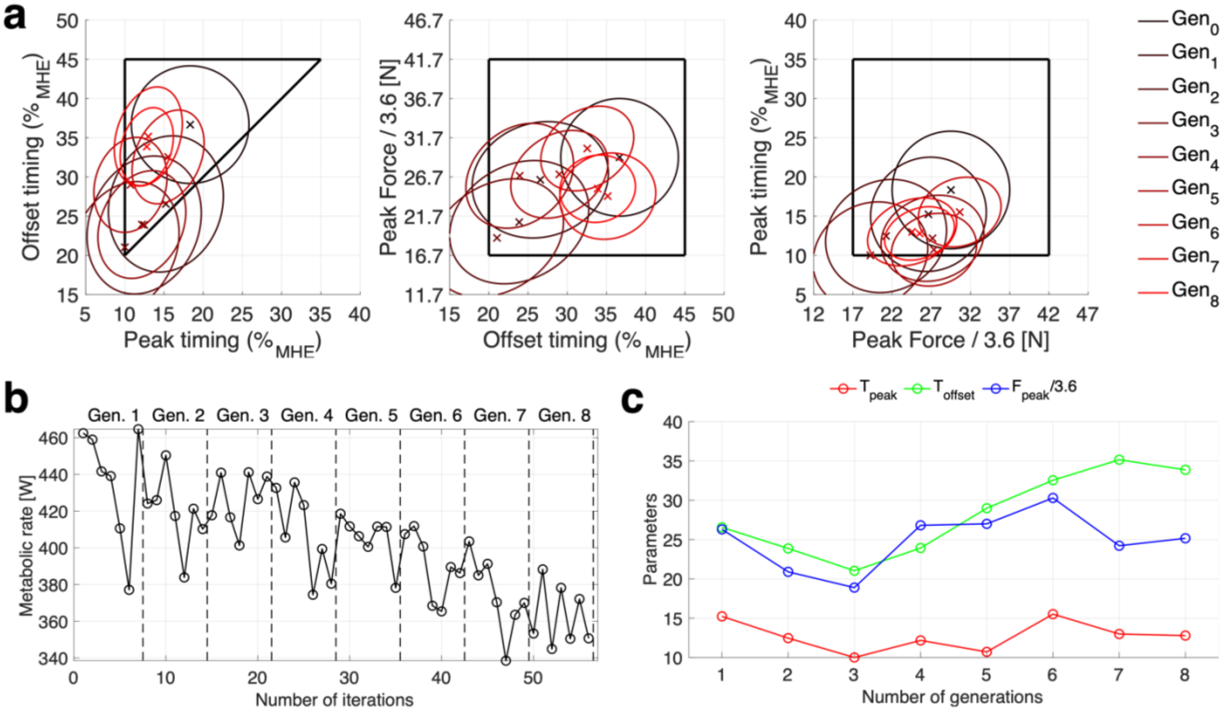

Figure S2. Experimental Result of the CMA-ES Optimization Process for a Representative Participant (P6). (**a**) The means and covariance matrices (i.e., ellipsoid) projection to the two-dimensional parameter subspace at each generation. The mean is marked as 'x'. The solid line means the covariance matrix. (**b**) The objective function evaluation (i.e., metabolic rate estimation) at each iteration. (**c**) The progress of the optimal point found by the CMA-ES algorithm after each generation.

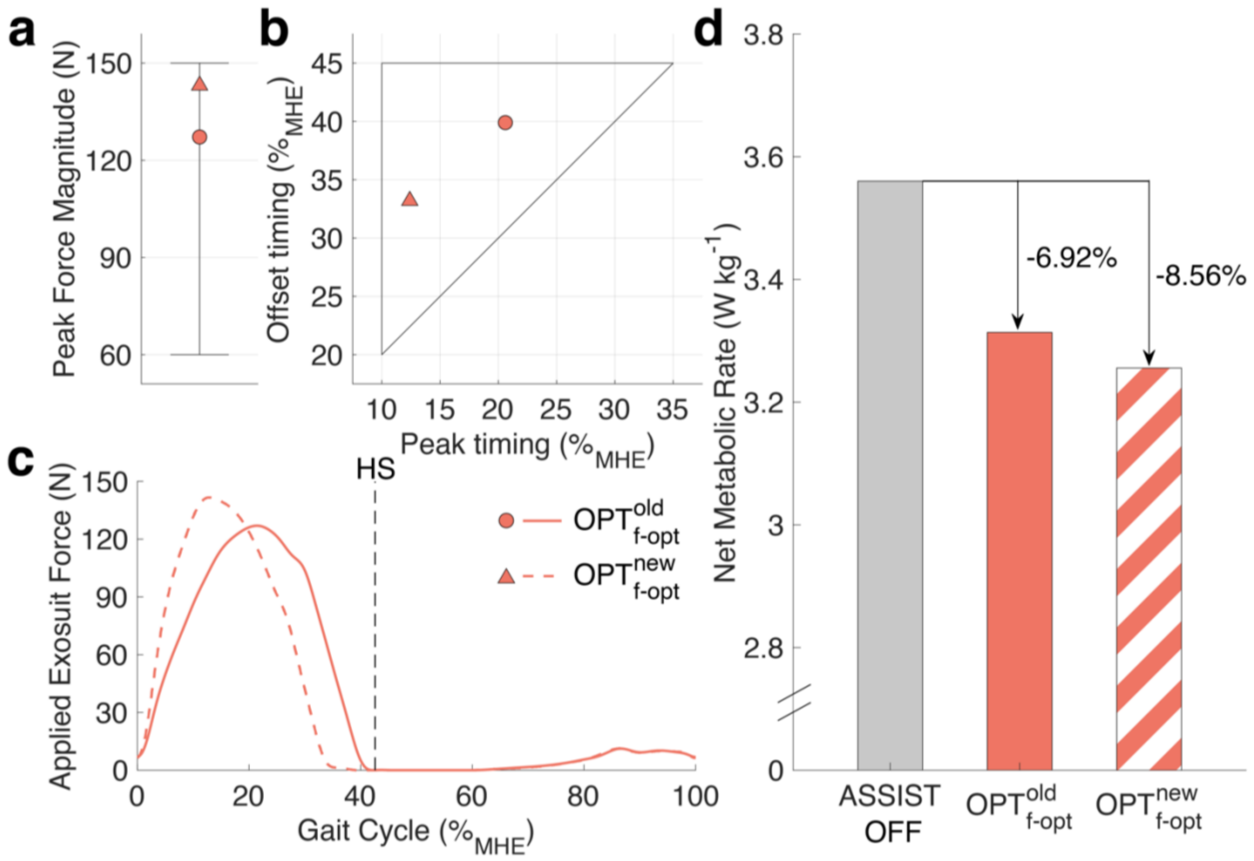

Figure S3. Repeating Optimization. (**a** and **b**) Both the old ($OPT_{f\text{-}opt}^{old}$) and new ($OPT_{f\text{-}opt}^{new}$) optimal profiles in the parameter space and (**c**) optimal force profiles across the gait cycle, segmented by MHE. The vertical dashed line represents a heel strike. The old optimal profile is the optimal profile identified on Optimization Day, and the new optimal profile is the optimal profile identified on Re-optimization Day for the same participant. (**d**) Net metabolic rates for $ASSIST\ OFF$ and both optimal parameter settings. (n = 1; P3 in Fig. 2 was recruited.)

Tables

Table S1. Participant Anthropometrics and Optimal Parameters. The age, mass, height, individualized control parameters identified by HIL optimization on Optimization Day, and optimal peak force identified by one dimensional (1D) validation on Biomechanics Day for each of the eight participants. The optimal force magnitude from 1D validation was chosen from the best net metabolic rate across the force sweep conditions ($OPT_{f\text{-}low}$, $OPT_{f\text{-}med}$, $OPT_{f\text{-}high}$, and $OPT_{f\text{-}max}$) in Supplementary Table S3. *Two conditions ($OPT_{f\text{-}med}$ and $OPT_{f\text{-}high}$) were tied for one participant (P6), and the average between the two was chosen as the optimal force magnitude. SD is the standard deviation.

	**Anthropometrics**			**HIL Optimization**			**1D Validation**
	Age	Mass (kg)	Height (cm)	Peak Timing (10–35%MHE)	Offset Timing (20–45%MHE)	Peak Force (60–150 N)	Peak Force (60, 90, 120 or 150 N)
Participant 1	30	58	170	15.9	40.8	77.9	60
Participant 2	29	60	168	13.0	32.7	70.3	90
Participant 3	29	93	183	20.6	39.9	127.2	60
Participant 4	31	77	178	15.3	43.2	66.9	120
Participant 5	50	82	180	17.4	33.9	126.9	90
Participant 6	28	79	178	12.8	33.9	90.6	105*
Participant 7	36	80	185	25.4	41.5	80.1	90
Participant 8	28	61	169	23.1	43.0	70.6	90
**Mean ± SD**	**32.6 ± 7.5**	**73.8 ± 12.6**	**176.4 ± 6.6**	**17.9 ± 4.7**	**38.6 ± 4.4**	**88.8 ± 24.7**	**88.1 ± 20.3**

Table S2. Metabolic Measurements Optimization Day (Day 2). The metabolic rate measurements in W kg^{-1} from Optimization Day including standing and the net metabolic rate (with standing subtracted) for the optimal ($OPT_{f\text{-}opt}$), a fixed profile ($MUS_{f\text{-}high}$), and two repeated $ASSIST\ OFF$ conditions. SEM is the standard error of the mean.

	Standing Metabolic Rate (W kg^{-1})	**Net Metabolic Rate (W kg^{-1})**			
		$OPT_{f\text{-}opt}$	$MUS_{f\text{-}high}$	$ASSIST\ OFF$ 1	$ASSIST\ OFF$ 2
Participant 1	1.37	3.93	4.89	5.33	4.95
Participant 2	2.14	2.73	2.98	3.57	3.53
Participant 3	1.26	2.93	3.19	3.27	3.46
Participant 4	1.71	2.86	2.94	3.60	3.45
Participant 5	0.96	2.60	2.60	3.36	3.10
Participant 6	1.70	2.54	2.47	2.71	2.64
Participant 7	1.43	3.08	3.42	3.20	3.26
Participant 8	1.46	2.59	3.49	3.14	2.86
**Mean ± SEM**	**1.50 ± 0.12**	**2.91 ± 0.16**	**3.25 ± 0.27**	**3.52 ± 0.28**	**3.41 ± 0.25**

Table S3. Metabolic Measurements Biomechanics Day (Day 3). The metabolic rate measurements in W kg^{-1} from Biomechanics Day including standing and the net metabolic rate (with standing subtracted) for all conditions tested. Conditions include optimal profile ($OPT_{f\text{-}opt}$), a fixed profile ($MUS_{f\text{-}high}$), optimal timing at four different fixed force magnitudes ($OPT_{f\text{-}low}$, $OPT_{f\text{-}med}$, $OPT_{f\text{-}high}$, $OPT_{f\text{-}max}$), two biologically inspired timings at the optimal force magnitude ($MUS_{f\text{-}opt}$, $TOR_{f\text{-}opt}$), $ASSIST\ OFF$, and two $NO\ EXO$ conditions. SEM is the standard error of the mean.

	Standing Metabolic Rate (W kg^{-1})	Net Metabolic Rate (W Kg^{-1})										
		$OPT_{f\text{-}opt}$	$MUS_{f\text{-}high}$	$OPT_{f\text{-}low}$	$OPT_{f\text{-}med}$	$OPT_{f\text{-}high}$	$OPT_{f\text{-}max}$	$MUS_{f\text{-}opt}$	$TOR_{f\text{-}opt}$	$ASSIST\ OFF$	$NO\ EXO$ 1	$NO\ EXO$ 2
Participant 1	1.92	3.46	4.45	3.43	3.64	3.88	3.93	3.66	3.60	3.75	3.41	3.70
Participant 2	2.12	3.11	3.89	3.56	3.09	3.26	3.93	4.25	3.36	3.05	3.04	3.45
Participant 3	1.54	2.56	2.85	2.48	2.53	2.82	2.85	2.77	2.66	2.66	3.09	2.90
Participant 4	1.46	2.99	3.27	3.34	3.20	2.87	3.11	3.47	3.51	3.59	3.08	3.13
Participant 5	0.80	2.51	2.48	2.32	2.29	2.95	2.50	2.84	2.66	2.74	2.67	2.88
Participant 6	1.82	3.05	3.37	3.47	3.31	3.31	3.40	3.15	3.27	3.22	3.53	3.31
Participant 7	1.48	3.21	4.39	3.63	3.46	3.56	4.02	3.71	4.08	3.70	3.45	3.78
Participant 8	1.68	3.50	4.13	3.97	3.78	4.39	4.53	3.68	4.00	4.56	4.23	3.90
**Mean ± SEM**	**1.60 ± 0.14**	**3.05 ± 0.13**	**3.60 ± 0.26**	**3.28 ± 0.20**	**3.16 ± 0.18**	**3.38 ± 0.19**	**3.53 ± 0.24**	**3.44 ± 0.18**	**3.39 ± 0.19**	**3.41 ± 0.22**	**3.31 ± 0.16**	**3.38 ± 0.14**

Table S4. Delivered Exosuit Torque and Power. Peak Exosuit Torque and Average Unilateral Positive and Negative Exosuit Power measured on the Biomechanics Day for each of the assist-on conditions, averaged across all participants.

	$OPT_{f\text{-}opt}$	$MUS_{f\text{-}high}$	$OPT_{f\text{-}low}$	$OPT_{f\text{-}med}$	$OPT_{f\text{-}high}$	$OPT_{f\text{-}max}$	$MUS_{f\text{-}opt}$	$TOR_{f\text{-}opt}$
Peak Exosuit Torque (Nm kg^{-1})	0.146	0.189	0.098	0.148	0.193	0.244	0.139	0.141
Average Unilateral Positive Exosuit Power (W kg^{-1})	0.077	0.104	0.052	0.079	0.108	0.144	0.074	0.047
Average Unilateral Negative Exosuit Power (W kg^{-1})	-0.008	-0.012	-0.007	-0.009	-0.010	-0.013	-0.009	-0.021

Table S5. Results for the Linear Regression of the Effect of Assistance Magnitude on Biomechanical Parameters (Investigation 2). Linear regression of the effect of assistance magnitude across all parameters analyzed ($y = m|\tau\ hip\ flex| + b$; y = biomechanics parameter; m = Linear Regression Slope; $|\tau\ hip\ flex|$ = absolute value of the average peak hip flexion torque; b = y intercept which is not presented for simplicity). Regression used data from the $ASSIST\ OFF$, $OPT_{f\text{-}low}$, $OPT_{f\text{-}med}$, $OPT_{f\text{-}high}$, and $OPT_{f\text{-}max}$ conditions. Abbreviations: Flex. = Flexion; Ext. = Extension; DF = Dorsiflexion; PF = Plantarflexion; Vel. = Velocity; Avg. = Average; Tor. = Torque; Pow. = Power; Bio. = Biological; Pos. = Positive; Neg. = Negative.

Parameter	Unit	Linear Regression Slope (m)	P value for slope	R^2
Stride Length	m	0.173	3.5×10^{-3}	0.93
Stride Time	s	0.139	3.5×10^{-3}	0.93
Cadence	steps s^{-1}	-0.111	2.0×10^{-3}	0.94
Max Hip Flex. Angle	degrees	53.123	1.3×10^{-6}	0.82
Max Hip Ext. Angle	degrees	11.907	4.9×10^{-3}	0.98
Max Hip Flex. Vel.	rad s^{-1}	5.604	1.4×10^{-11}	0.92
Max Hip Ext. Vel.	rad s^{-1}	-2.603	3.0×10^{-4}	0.69
Max Knee Flex. Angle	degrees	34.162	3.0×10^{-4}	0.72
Max Knee Ext. Angle	degrees	11.977	2.2×10^{-2}	0.94
Max Knee Flex. Vel.	rad s^{-1}	3.229	1.1×10^{-4}	0.88
Max Knee Ext. Vel.	rad s^{-1}	0.019	9.8×10^{-1}	0.85
Max Ankle DF Angle	degrees	7.090	3.2×10^{-2}	0.95
Max Ankle PF Angle	degrees	10.332	7.8×10^{-3}	0.96
Max Ankle DF Vel.	rad s^{-1}	1.994	6.7×10^{-2}	0.83
Max Ankle PF Vel.	rad s^{-1}	2.673	3.2×10^{-5}	0.87
Max Hip Flex. Tor.	Nm kg^{-1}	0.134	1.7×10^{-1}	0.97
Max Hip Bio. Flex. Tor.	Nm kg^{-1}	0.147	1.3×10^{-1}	0.98
Max Hip Ext. Tor.	Nm kg^{-1}	0.078	6.2×10^{-1}	0.95
Max Knee Flex. Tor.	Nm kg^{-1}	0.133	1.2×10^{-2}	0.85
Max Knee Ext. Tor.	Nm kg^{-1}	0.105	3.6×10^{-1}	0.99
Max Ankle DF Tor.	Nm kg^{-1}	-0.157	2.0×10^{-3}	0.87
Max Ankle PF Tor.	Nm kg^{-1}	-0.031	7.1×10^{-1}	0.96
Avg. Hip Flex. Tor.	Nm kg^{-1}	0.007	7.5×10^{-1}	0.98
Avg. Hip Bio. Flex. Tor.	Nm kg^{-1}	0.080	5.4×10^{-4}	0.98
Avg. Hip Ext. Tor.	Nm kg^{-1}	0.091	3.8×10^{-3}	0.98
Avg. Hip Bio Ext. Tor.	Nm kg^{-1}	0.238	4.3×10^{-8}	0.98
Avg. Knee Flex. Tor.	Nm kg^{-1}	0.014	3.9×10^{-1}	0.99
Avg. Knee Ext. Tor.	Nm kg^{-1}	0.007	7.7×10^{-1}	0.99
Avg. Ankle DF Tor.	Nm kg^{-1}	-0.016	6.4×10^{-3}	0.91
Avg. Ankle PF Tor.	Nm kg^{-1}	-0.146	5.2×10^{-4}	0.95

Max Hip Pos. Pow.	W kg^{-1}	3.506	3.9×10^{-8}	0.92
Max Hip Neg. Pow.	W kg^{-1}	-0.909	1.6×10^{-3}	0.91
Max Hip Pos. Bio. Pow.	W kg^{-1}	2.106	4.6×10^{-4}	0.75
Max Hip Neg. Bio. Pow.	W kg^{-1}	-1.385	3.6×10^{-6}	0.91
Max Knee Pos. Pow.	W kg^{-1}	-1.503	1.4×10^{-3}	0.69
Max Knee Neg. Pow.	W kg^{-1}	-0.091	7.9×10^{-1}	0.89
Max Ankle Pos. Pow.	W kg^{-1}	0.372	4.9×10^{-1}	0.93
Max Ankle Neg. Pow.	W kg^{-1}	-1.696	7.5×10^{-3}	0.84
Avg. Hip Pos. Pow.	W kg^{-1}	0.440	4.3×10^{-6}	0.93
Avg. Hip Neg. Pow.	W kg^{-1}	-0.062	4.6×10^{-3}	0.98
Avg. Hip Pos. Bio. Pow.	W kg^{-1}	0.133	1.0×10^{-1}	0.90
Avg. Hip Neg. Bio. Pow.	W kg^{-1}	-0.348	5.7×10^{-11}	0.97
Avg. Knee Pos. Pow.	W kg^{-1}	-0.128	1.3×10^{-3}	0.87
Avg. Knee Neg. Pow.	W kg^{-1}	-0.070	3.5×10^{-2}	0.99
Avg. Ankle Pos. Pow.	W kg^{-1}	0.001	9.8×10^{-1}	0.96
Avg. Ankle Neg. Pow.	W kg^{-1}	-0.025	3.9×10^{-1}	0.97

Table S6. Biomechanical Results for $ASSIST\ OFF$, $NO\ EXO$, and Optimal Timing vs. Biologically Inspired Timings (Investigation 3). Biomechanical results (mean ± SEM) across all parameters analyzed for the $ASSIST\ OFF$, $NO\ EXO$, $OPT_{f\text{-}opt}$, $TOR_{f\text{-}opt}$, and $MUS_{f\text{-}opt}$ conditions. We compared (i) all conditions to the $ASSIST\ OFF$ condition and (ii) $OPT_{f\text{-}opt}$ to the two other active conditions ($MUS_{f\text{-}opt}$ and $TOR_{f\text{-}opt}$) using repeated measures ANOVA. *Significant difference relative to $ASSIST\ OFF$, $P < 0.05$. #Significant difference relative to $OPT_{f\text{-}opt}$, $P < 0.05$ ($MUS_{f\text{-}opt}$ and $TOR_{f\text{-}opt}$ only) Abbreviations: Flex. = Flexion; Ext. = Extension; DF = Dorsiflexion; PF = Plantarflexion; Vel. = Velocity; Avg. = Average; Tor. = Torque; Pow. = Power; Bio. = Biological; Pos. = Positive; Neg. = Negative.

Parameter	Unit	$ASSIST\ OFF$	$NO\ EXO$	$OPT_{f\text{-}opt}$	$MUS_{f\text{-}opt}$	$TOR_{f\text{-}opt}$
Stride Length	m	1.36 ± 0.01	1.37 ± 0.00	1.40 ± 0.01*	1.39 ± 0.01	1.38 ± 0.01
Stride Time	s	1.09 ± 0.00	1.09 ± 0.00	1.12 ± 0.01*	1.11 ± 0.01	1.10 ± 0.00
Cadence	steps s^{-1}	0.92 ± 0.00	0.92 ± 0.00	0.89 ± 0.00	0.90 ± 0.00	0.91 ± 0.00
Max Hip Flex. Angle	degrees	32.3 ± 0.8	33.6 ± 0.7*	39.6 ± 0.8*	39.7 ± 0.9*	36.4 ± 0.8*#
Max Hip Ext. Angle	degrees	-9.8 ± 0.9	-9.4 ± 1.0	-9.1 ± 0.9	-8.8 ± 0.9	-6.0 ± 1.0#
Max Hip Flex. Vel.	rad s^{-1}	3.37 ± 0.04	3.54 ± 0.05*	4.03 ± 0.04*	3.94 ± 0.03*	3.45 ± 0.04*#
Max Hip Ext. Vel.	rad s^{-1}	-2.02 ± 0.02	-2.08 ± 0.02	-2.19 ± 0.03	-2.16 ± 0.03	-2.32 ± 0.03
Max Knee Flex. Angle	degrees	72.1 ± 0.3	73.4 ± 0.4*	76.6 ± 0.3*	76.6 ± 0.3*	74.1 ± 0.4*#
Max Knee Ext. Angle	degrees	0.0 ± 0.6	0.2 ± 0.6	-0.2 ± 0.7	0.8 ± 0.7	0.1 ± 0.6
Max Knee Flex. Vel.	rad s^{-1}	6.63 ± 0.05	6.71 ± 0.06	6.70 ± 0.05	6.64 ± 0.05	6.91 ± 0.03
Max Knee Ext. Vel.	rad s^{-1}	-7.63 ± 0.09	-7.89 ± 0.09*	-8.39 ± 0.09*	-8.09 ± 0.08*#	-7.73 ± 0.09*#
Max Ankle DF Angle	degrees	14.1 ± 0.6	14.6 ± 0.6	15.2 ± 0.5*	15.1 ± 0.5*	14.9 ± 0.5*
Max Ankle PF Angle	degrees	-17.5 ± 0.6	-16.7 ± 0.5	-17.0 ± 0.6	-17.0 ± 0.6	-17.5 ± 0.6
Max Ankle DF Vel.	rad s^{-1}	5.20 ± 0.09	5.25 ± 0.09	5.23 ± 0.10	5.23 ± 0.11	5.36 ± 0.11
Max Ankle PF Vel.	rad s^{-1}	-2.87 ± 0.07	-2.86 ± 0.05	-2.50 ± 0.06*	-2.54 ± 0.07*	-2.83 ± 0.06*#
Max Hip Flex. Tor.	Nm kg^{-1}	-0.598 ± 0.017	-0.599 ± 0.019	-0.552 ± 0.020*	-0.565 ± 0.019	-0.582 ± 0.018
Max Hip Bio. Flex. Tor.	Nm kg^{-1}	-0.598 ± 0.017	-0.599 ± 0.019	-0.525 ± 0.022*	-0.541 ± 0.020*	-0.459 ± 0.020*#
Max Hip Ext. Tor.	Nm kg^{-1}	0.826 ± 0.018	0.852 ± 0.019	0.842 ± 0.020	0.836 ± 0.020	0.861 ± 0.019
Max Knee Flex. Tor.	Nm kg^{-1}	-0.444 ± 0.005	-0.453 ± 0.004	-0.454 ± 0.005	-0.449 ± 0.003	-0.448 ± 0.005
Max Knee Ext. Tor.	Nm kg^{-1}	0.550 ± 0.035	0.610 ± 0.041*	0.543 ± 0.036	0.572 ± 0.037	0.556 ± 0.039
Max Ankle DF Tor.	Nm kg^{-1}	-0.174 ± 0.005	-0.174 ± 0.004	-0.189 ± 0.004	-0.186 ± 0.003	-0.176 ± 0.004
Max Ankle PF Tor.	Nm kg^{-1}	1.750 ± 0.013	1.716 ± 0.017	1.759 ± 0.014	1.756 ± 0.015	1.750 ± 0.013
Avg. Hip Flex. Tor.	Nm kg^{-1}	-0.115 ± 0.004	-0.114 ± 0.004	-0.110 ± 0.004	-0.113 ± 0.004	-0.118 ± 0.004#
Avg. Hip Bio. Flex. Tor.	Nm kg^{-1}	-0.115 ± 0.004	-0.114 ± 0.004	-0.088 ± 0.004*	-0.091 ± 0.005*	-0.081 ± 0.005*
Avg. Hip Ext. Tor.	Nm kg^{-1}	0.172 ± 0.007	0.172 ± 0.006	0.188 ± 0.007*	0.185 ± 0.007*	0.184 ± 0.007*
Avg. Hip Bio Ext. Tor.	Nm kg^{-1}	0.172 ± 0.007	0.172 ± 0.006	0.204 ± 0.008*	0.202 ± 0.007*	0.190 ± 0.008*#
Avg. Knee Flex. Tor.	Nm kg^{-1}	-0.099 ± 0.004	-0.096 ± 0.005	-0.102 ± 0.005	-0.097 ± 0.005	-0.095 ± 0.005
Avg. Knee Ext. Tor.	Nm kg^{-1}	0.095 ± 0.007	0.103 ± 0.008	0.094 ± 0.008	0.101 ± 0.008	0.101 ± 0.008

Avg. Ankle DF Tor.	Nm kg^{-1}	-0.017 ± 0.001	-0.017 ± 0.001	-0.018 ± 0.001	-0.018 ± 0.000	-0.017 ± 0.001
Avg. Ankle PF Tor.	Nm kg^{-1}	0.415 ± 0.006	0.412 ± 0.006	0.396 ± 0.006*	0.394 ± 0.005*	0.407 ± 0.006$^{*#}$
Max Hip Pos. Pow.	W kg^{-1}	1.015 ± 0.030	1.036 ± 0.025	1.22 ± 0.042*	1.22 ± 0.029	1.175 ± 0.032
Max Hip Neg. Pow.	W kg^{-1}	-0.452 ± 0.018	-0.534 ± 0.017*	-0.523 ± 0.028	-0.583 ± 0.028$^{*#}$	-0.466 ± 0.020*
Max Hip Pos. Bio. Pow.	W kg^{-1}	1.015 ± 0.03	1.036 ± 0.025	1.161 ± 0.03	1.174 ± 0.028	1.054 ± 0.031
Max Hip Neg. Bio. Pow.	W kg^{-1}	-0.452 ± 0.018	-0.534 ± 0.017*	-0.548 ± 0.021*	-0.578 ± 0.025*	-0.399 ± 0.021$^{*#}$
Max Knee Pos. Pow.	W kg^{-1}	0.879 ± 0.030	0.907 ± 0.028	0.873 ± 0.039	0.810 ± 0.038	0.880 ± 0.032
Max Knee Neg. Pow.	W kg^{-1}	-1.811 ± 0.028	-1.901 ± 0.034	-2.115 ± 0.036*	-2.064 ± 0.032*	-1.952 ± 0.040*
Max Ankle Pos. Pow.	W kg^{-1}	3.625 ± 0.068	3.464 ± 0.069	3.681 ± 0.068	3.707 ± 0.074	3.724 ± 0.076
Max Ankle Neg. Pow.	W kg^{-1}	-1.442 ± 0.045	-1.438 ± 0.047	-1.631 ± 0.049*	-1.648 ± 0.054*	-1.499 ± 0.048*
Avg. Hip Pos. Pow.	W kg^{-1}	0.249 ± 0.010	0.25 ± 0.009	0.304 ± 0.010*	0.303 ± 0.009*	0.284 ± 0.011*
Avg. Hip Neg. Pow.	W kg^{-1}	-0.060 ± 0.005	-0.064 ± 0.005	-0.058 ± 0.005	-0.063 ± 0.005	-0.061 ± 0.005
Avg. Hip Pos. Bio. Pow.	W kg^{-1}	0.249 ± 0.010	0.250 ± 0.009	0.250 ± 0.008	0.252 ± 0.008	0.244 ± 0.010
Avg. Hip Neg. Bio. Pow.	W kg^{-1}	-0.060 ± 0.005	-0.064 ± 0.005	-0.072 ± 0.005*	-0.077 ± 0.005*	-0.047 ± 0.005$^{*#}$
Avg. Knee Pos. Pow.	W kg^{-1}	0.110 ± 0.004	0.113 ± 0.005	0.107 ± 0.004	0.103 ± 0.004	0.109 ± 0.004
Avg. Knee Neg. Pow.	W kg^{-1}	-0.266 ± 0.008	-0.276 ± 0.006	-0.285 ± 0.010	-0.292 ± 0.010*	-0.282 ± 0.010*
Avg. Ankle Pos. Pow.	W kg^{-1}	0.251 ± 0.005	0.236 ± 0.005*	0.250 ± 0.005	0.252 ± 0.004	0.258 ± 0.006
Avg. Ankle Neg. Pow.	W kg^{-1}	-0.251 ± 0.006	-0.247 ± 0.005	-0.253 ± 0.006	-0.252 ± 0.006	-0.252 ± 0.007

Table S7. Metabolic Measurements – Autonomous and Portable Exosuit Testing. The metabolic rate measurements in W kg^{-1} from the autonomous system testing including standing and the net metabolic rate (with standing subtracted) for all conditions tested. Conditions include the optimal profile ($OPT_{f\text{-}opt}$) and $NO\ EXO$ conditions, each repeated twice to account for metabolic noise. SEM is the standard error of the mean.

	Standing Metabolic Rate (W kg^{-1})	**Net Metabolic Rate (W kg^{-1})**			
		$OPT_{f\text{-}opt}$ 1	$OPT_{f\text{-}opt}$ 2	$NO\ EXO$ 1	$NO\ EXO$ 2
Participant 2	1.49	2.97	3.03	3.29	3.49
Participant 7	1.16	2.50	2.56	2.45	2.69
Participant 8	1.52	2.75	2.89	3.05	3.13
**Mean ± SEM**	**1.39 ± 0.12**	**2.74 ± 0.14**	**2.83 ± 0.14**	**2.93 ± 0.25**	**3.10 ± 0.23**

Table S8. Apparent joint efficiency – hip extension. Studies were included if they met the following criteria: (i) used an active, bilateral exoskeleton to independently assist hip extension; (ii) demonstrated a metabolic reduction relative to powered off; and (iii) provided a bilateral sum of average positive exoskeleton power. Apparent joint efficiency was calculated as a ratio of the average bilateral positive exoskeleton power to the metabolic reduction. SEM is the standard error of the mean.

	Average Bilateral Positive Exoskeleton Power (W kg^{-1})	Metabolic Impact Relative to Powered Off (W kg^{-1})	Apparent Joint Efficiency
LSEP[27]	0.178	-0.370	0.481
ESEP[27]	0.206	-0.350	0.589
ESLP[27]	0.227	-0.500	0.454
LSLP[27]	0.205	-0.420	0.489
Hip-only[28]	0.089	-0.210	0.424
Session 3 [29]	0.320	-0.630	0.507
Session 4 [29]	0.316	-0.565	0.559
Session 5 [29]	0.332	-0.584	0.569
Walking[1]	0.250	-0.657	0.381
**Mean ± SEM**	**0.236 ± 0.026**	**-0.476 ± 0.050**	**0.495 ± 0.023**

Table S9. Apparent joint efficiency – ankle plantarflexion. Studies were included if they met the following criteria: (i) used an active, bilateral exoskeleton to independently assist ankle plantarflexion; (ii) demonstrated a metabolic reduction relative to powered off; and (iii) provided a bilateral sum of average positive exoskeleton power. Apparent joint efficiency was calculated as a ratio of the average bilateral positive exoskeleton power to the metabolic reduction. SEM is the standard error of the mean.

	Average Bilateral Positive Exoskeleton Power (W kg^{-1})	Metabolic Impact Relative to Powered Off (W kg^{-1})	Apparent Joint Efficiency
Powered end Session 3 [16]	0.240	-0.320	0.750
Preferred step length[30]	0.230	-0.390	0.590
43% onset[31]	0.136	-0.640	0.213
[32]	0.306	-0.580	0.527
Session 3 [33]	0.380	-0.650	0.585
[34]	0.210	-0.540	0.389
Early-Medium[35]	0.420	-0.870	0.483
Optimized[36]	0.300	-1.210	0.248
Ankle-only[37]	0.682	-1.120	0.609
**Mean ± SEM**	**0.323 ± 0.053**	**-0.702 ± 0.102**	**0.488 ± 0.059**

Video legends

Video S1. A participant waking with hip flexion exosuit with optimization algorithm running (Optimization Day).

Video S2. A participant walking with hip flexion exosuit with metabolic, kinematics, kinetics, and electromyography measurements (Biomechanics Day).

Video S3. An autonomous and portable exosuit in indoor (Supplemental study) and outdoor environments.
